# Identification of Novel 3-Hydroxy-pyran-4-One Derivatives as Potent HIV-1 Integrase Inhibitors Using *in silico* Structure-Based Combinatorial Library Design Approach

**DOI:** 10.3389/fchem.2019.00574

**Published:** 2019-08-13

**Authors:** Hajar Sirous, Giulia Chemi, Sandra Gemma, Stefania Butini, Zeger Debyser, Frauke Christ, Lotfollah Saghaie, Simone Brogi, Afshin Fassihi, Giuseppe Campiani, Margherita Brindisi

**Affiliations:** ^1^Bioinformatics Research Center, School of Pharmacy and Pharmaceutical Sciences, Isfahan University of Medical Sciences, Isfahan, Iran; ^2^Department of Biotechnology, Chemistry and Pharmacy, Department of Excellence 2018-2022, University of Siena, Siena, Italy; ^3^Molecular Medicine, K.U. Leuven and IRC KULAK, Leuven, Belgium; ^4^Department of Medicinal Chemistry, Faculty of Pharmacy, Isfahan University of Medical Sciences, Isfahan, Iran; ^5^Department of Pharmacy, University of Pisa, Pisa, Italy; ^6^Department of Pharmacy, Department of Excellence 2018-2022, University of Naples Federico II, Naples, Italy

**Keywords:** 3-hydroxy-pyran-4-one, HIV-1 integrase inhibitors (HIV-1 INIs), *in silico* combinatorial library design, side chain hopping, hit compounds optimization

## Abstract

We describe herein the development and experimental validation of a computational protocol for optimizing a series of 3-hydroxy-pyran-4-one derivatives as HIV integrase inhibitors (HIV INIs). Starting from a previously developed micromolar inhibitors of HIV integrase (HIV IN), we performed an in-depth investigation based on an *in silico* structure-based combinatorial library designing approach. This method allowed us to combine a combinatorial library design and side chain hopping with Quantum Polarized Ligand Docking (QPLD) studies and Molecular Dynamics (MD) simulation. The combinatorial library design allowed the identification of the best decorations for our promising scaffold. The resulting compounds were assessed by the mentioned QPLD methodology using a homology model of full-length binary HIV IN/DNA for retrieving the best performing compounds acting as HIV INIs. Along with the prediction of physico-chemical properties, we were able to select a limited number of drug-like compounds potentially displaying potent HIV IN inhibition. From this final set, based on the synthetic accessibility, we further shortlisted three representative compounds for the synthesis. The compounds were experimentally assessed *in vitro* for evaluating overall HIV-1 IN inhibition, HIV-1 IN strand transfer activity inhibition, HIV-1 activity inhibition and cellular toxicity. Gratifyingly, all of them showed relevant inhibitory activity in the *in vitro* tests along with no toxicity. Among them **HPCAR-28** represents the most promising compound as potential anti-HIV agent, showing inhibitory activity against HIV IN in the low nanomolar range, comparable to that found for Raltegravir, and relevant potency in inhibiting HIV-1 replication and HIV-1 IN strand transfer activity. In summary, our results outline **HPCAR-28** as a useful optimized hit for the potential treatment of HIV-1 infection by targeting HIV IN.

## Introduction

HIV-1 integrase (IN) represents an attractive target in anti-HIV drug design mainly due to its specificity. Accordingly, HIV-1 IN does not have a functional equivalent in humans and plays a unique role in establishing irreversible and productive viral infections (Debyser et al., 2002; Delelis et al., 2008). This viral key enzyme catalyzes the insertion of proviral DNA, derived from reverse transcription of HIV-1 RNA, into the genome of the host-infected cells. The insertion is achieved through a two-step enzymatic process which starts with endonucleolytic cleavage of a terminal dinucleotide (GT) from each 3′-end of the proviral DNA (termed “3′-processing”), followed by a second reaction, known as “strand transfer” (ST), involving a concerted nucleophilic attack, by the reactive 3′-OH ends of the viral processed DNA to the host chromosomal DNA. As a result, a the covalent joining of the two DNA strands is observed (Chiu and Davies, 2004; Pommier et al., 2005). Both reactions are accomplished by the catalytic core domain of HIV-1 IN which contains two divalent metal ion cofactors (Mg^2+^). These metal ions are coordinated by three catalytic carboxylate residues: Asp_64_, Asp_116_, and Glu_152_ (DDE triad) within the enzyme active site (Dyda et al., 1994; Neamati et al., 2002).

**Figure 1:**
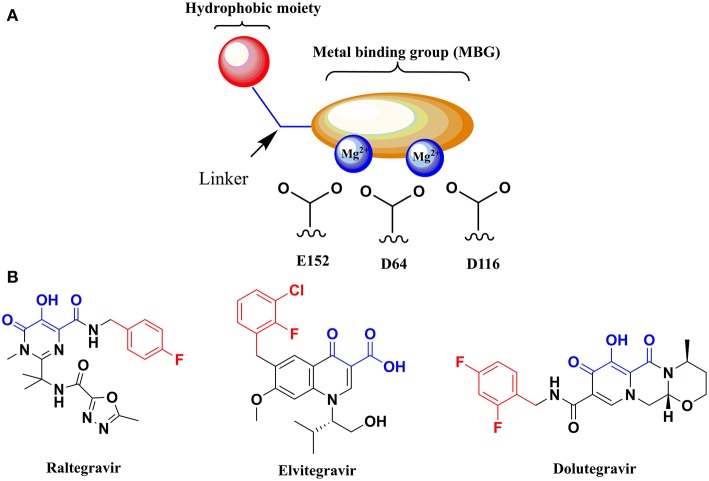
**(A)** Graphical depiction of the pharmacophore model for HIV-1 INIs. **(B)** Chemical structure of the FDA approved HIV-1 INIs. Atoms in blue are part of MBG of the molecules able to chelate the two metal ions. The hydrophobic aromatic moiety of each compound is highlighted in red.

Targeting the metal cofactors within the active site of a viral metal-activated enzyme like HIV-1 IN has emerged as an attractive and validated strategy for the development of novel anti-HIV agents (Rogolino et al., 2012). With this aim, a metal binding pharmacophore model has been exploited for the design of diverse HIV-1 integrase inhibitors (HIV-1 INIs) as depicted in Figure 1A. This model is represented by two distinctive structural features: (1) a planar metal binding group (MBG), able to interact with the metal centers within the IN active site, and (2) a pendent aromatic or hetero-aromatic hydrophobic moiety located in close proximity of the MBG (Kawasuji et al., 2006a,b; Johns and Svolto, 2008). Continuous efforts in exploiting this pharmacophore model have culminated in the design and subsequent FDA approval of three INIs for clinical use as effective anti-HIV drugs: Raltegravir (**RLT**), Elvitegravir (**EVG**), and Dolutegravir (**DTG**) in 2007, 2012, and 2013, respectively (Figure 1B; Rowley, 2008; Sato et al., 2009; Katlama and Murphy, 2012).

**Figure 2:**
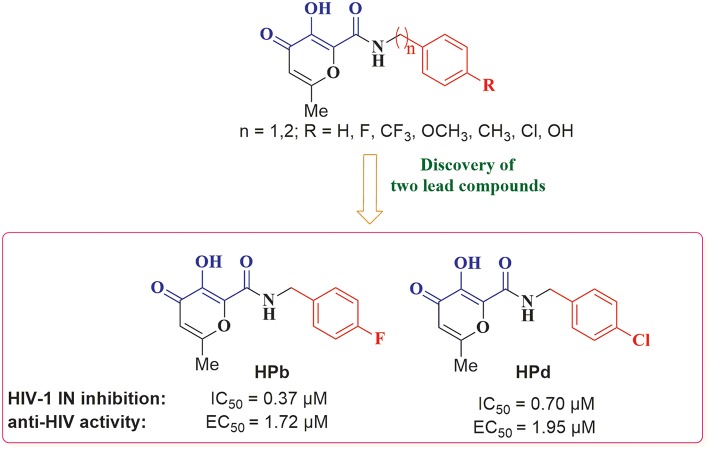
General structure of 3-hydroxy-4-pyranone (HP) based inhibitors previously designed in our laboratory and two halogenated derivatives identified as lead compounds. The proposed chemotype satisfies minimal pharmacophore features for HIV-1 IN inhibition: the metal chelating triad (blue) and terminal hydrophobic benzyl group (red). The most promising HP compounds are shown with the IC_50_ and EC_50_ values for IN inhibitory and anti-HIV activities.

A variety of MBGs have been extensively studied to design innovative and effective INIs (Liao et al., 2010; Di Santo, 2014). Recently, we were particularly interested in taking advantage of the 3-hydroxy-4-pyranone (HP) scaffold for the development of novel HIV-1 INIs due to its application as MBG in the design of several inhibitors of numerous Zn^2+^, Mg^2+^, Mn^2+^, and Cu^2+^ dependent proteins. Accordingly, HP derivatives represent an impressive class of heterocyclic ligands with strong bidentate chelating capacity toward metal ions (Santos et al., 2012; Rostami et al., 2015; Sirous et al., 2015). As a first example of the potential of this structural template in HIV-IN inhibition, a series of HP compounds featuring a unique C-2 carboxamide moiety, namely 3-hydroxyl-pyran-4-one-2-carboxamide derivatives (HPCARs), were rationally designed and recently reported by us (Figure 2; Sirous et al., 2019). The proposed chemotypes were characterized by a chelating triad motif effectively coordinating the two metals according to the pharmacophore shared by INIs. Moreover, an aromatic backbone attached to the amide portion through a linker (substituted benzyl and phenylethyl moieties) was considered for providing the essential interactions with the hydrophobic pocket of the enzyme. Most of these HPCAR analogs offered favorable inhibitory potencies in both enzymatic and cell-based antiviral assays with low micromolar IC_50_ values. In particular, the substitution at the *para* position of the aromatic phenyl ring led to the identification of two halo-benzyl derivatives **HPb** and **HPd** (Figure 2) as promising lead HIV-1 IN inhibitors with IC_50_ values of 0.37 and 0.7 μM, respectively (Sirous et al., 2019).

**Figure 3:**
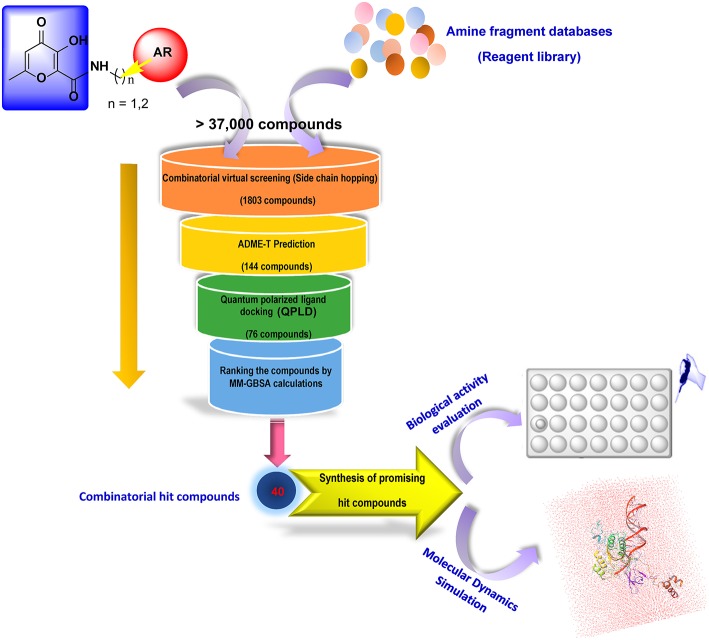
Screening workflow employed in the present study for the *in silico* rational design of new HPCAR derivatives. The core of HPCAR was marked in a blue box. The hydrophobic fragment, AR group, is depicted as a red circle. During combinatorial library screening, diverse sets of fragments were provided from reagent file libraries and attached to the main core at the defined attach positions. The attach position in the scaffold structure is indicated as a yellow vector.

In our quest for the search of innovative and effective INIs and considering the above-mentioned findings, we decided to design novel optimized derivatives exploiting the HPCAR chemotype. In this study, we performed the replacement of the pendant aromatic portion with other heterocyclic moieties in order to maintain the strong hydrophobic interactions within the HIV-1 IN binding site, with the possibility to explore additional functional groups for maximizing the contacts that could further stabilize the binding mode of the novel derivatives, leading to compounds with improved activity against HIV-1 IN. Accordingly, in the present study, an *in silico* protocol combining a combinatorial library design procedure coupled to extensive molecular docking studies and physico-chemical properties prediction was developed in a step-filtering approach to identify novel INIs with improved potency with respect to the HPCAR derivatives. The employed screening workflow for designing new HIV-1 INIs with suitable potency and satisfactory physico-chemical properties is illustrated in Figure 3. Considering the importance of the aromatic portion of INIs for their binding to both the viral DNA bases, and the hydrophobic pocket within the catalytic core of IN enzyme (Kawasuji et al., 2006b), many efforts were made for replacing the hydrophobic aromatic side chains (substituted benzyl and phenylethyl moieties) to generate a virtual combinatorial library of HP-based core derivatives. Accordingly, using side chain hopping strategy, various cyclic and heterocyclic fragments were attached to the defined position of the HP core in order to find the ideal sidechains with the highest predicted binding affinity for the IN active site. Finally, for validating the computational approach three representative hit candidates identified from this screening workflow were selected, further studied by molecular dynamics (MD) simulations in order to gain additional information about their mechanism of action as INIs, synthesized and submitted to biological evaluation for their HIV-1 IN inhibitory and anti-HIV-1 activities.

## Materials and Methods

### Computational Details

#### Ligand Preparation

The 3D structure of the two investigated HPCAR derivatives (substituted with benzyl and phenylethyl moieties) were built by the 3D-sketcher module in Maestro suite (Maestro, version 9.2; Schrödinger, LLC, New York, NY, 2011). Molecular energy minimization of the structures was performed in MacroModel environment using the OPLS-AA 2005 as force field (Jorgensen et al., 1996; Kaminski et al., 2001). GB/SA model was utilized in order to simulate the solvent effects applying “no cut-off” for non-bonded interactions (Still et al., 1990). PRCG method with 1,000 maximum iterations and 0.001 gradient convergence threshold was employed. The same protocol was applied to the novel designed compounds obtained by the combinatorial screening (144 molecules) before submitting them to QPLD procedure. Furthermore, all the compounds were accurately prepared with LigPrep application implemented in Maestro suite (Gasser et al., 2015). Finally the most probable ionization state of the compounds was retrieved by Chemicalize (https://chemicalize.com/) as already reported by us (Brogi et al., 2018).

#### Protein Preparation

Computational studies were conducted using our recently described theoretical model of full-length HIV-1 IN in complex with viral DNA and Mg^2+^ cofactors (Sirous et al., 2019). The model was subjected to Protein Preparation Wizard protocol implemented in Maestro. This protocol allowed us to obtain a reasonable starting structure of the protein for molecular modeling calculations by a series of computational steps as described (Cappelli et al., 2013; Brogi et al., 2017a,b). Finally, the refined HIV-1 IN model was used for further computational studies.

#### Generation of Combinatorial Hits

“Combinatorial library enumeration” option available in CombiGlide (CombiGlide, version 2.7; Schrödinger, LLC: New York, 2011), a combinatorial screening software distributed by Schrödinger, was used to carry out structure-based combinatorial library design studies. This software provides the tools for accelerating the lead optimization process, helping in the generation of libraries of optimized derivatives to be selected for the further synthesis. In this direction, two HPCAR derivatives containing methylene and ethylene linkers between the chelating region and the aromatic moiety, identified from our previous studies (Sirous et al., 2019), were selected as main cores. For each investigated compound, a side chain hopping strategy was successfully applied for replacing the hydrophobic side chain, of the selected main cores, with different aromatic or heteroaromatic fragments as shown in Figure 3. This method employs the reagent files, chosen by the operator, as a source of fragments with various structures. The following steps are used to generate a new combinatorial library of ligands.

#### Reagents Preparation

In this step of CombiGlide workflow, a library of reagents containing diverse sets of fragments was built. The elements of this library can be selected from the available databases or generated by the operator. In fact, in the presented work, in addition to the reagent libraries provided by Maestro software, other reagent libraries with different aromatic groups were downloaded from Zinc fragment database (Irwin and Shoichet, 2005; Irwin et al., 2012) as SDF file format and submitted to the reagent preparation facility in the CombiGlide environment. Then the tasks in the reagent preparation process are: (i) the selection of the source of reagent structures; (ii) the selection of a reagent type (a functional group), and finally (iii) the structural conversion from the 2D structure to the 3D one. The selection of a reagent type was done considering the bond that will be replaced in accordance to the functional group formed when the reagent is added to the core. In this context, primary amine set was selected as reagent type. The detailed description of this reagent type is provided in Table 1. Concerning this reagent type, R represents the part of reagent that was kept in the process of combinatorial library generation. The bond that was broken to attach the reagent to the core was marked with a line crossing the bond. After running reagent preparation job, the output structure file in .bld format, containing properly prepared reagents, was used in the combinatorial screening process by CombiGlide.

**Table 1:** Detailed description of predefined functional group type selected in the reagents preparation step of combinatorial library design.

**Structure of reagent type**	**Name of reagent type**	**Definition of R**
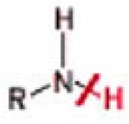	Amine_Primary_General_N_H	R can be alkyl, aryl. R cannot have a carbonyl carbon attached to the nitrogen of the amine.

#### Defining the Core and Attachments

The core is the structural element that is maintained throughout the combinatorial experiment. The attachment positions for each core were defined and the previously prepared reagents file was associated with each attachment point. The attachment point comprises bonds from the core structure that will be replaced in the build process. Considered the role of the hydrophobic side chain of the investigated HPCAR derivatives in binding both the viral DNA and the hydrophobic pocket within IN active site (Sirous et al., 2019), the replacement of benzyl and phenethyl amine moieties with different amine fragments from the reagents file, with other hydrophobic moieties, was performed.

#### Setting Up CombiGlide Docking Calculations

The docking step represents the main step of the combinatorial screening process in which a series of docking calculations are performed to screen out molecules that do not have satisfactory docking scores. In fact, on a core with a constant structure, CombiGlide attaches sidechains at defined positions of the core, and performs a docking calculation of the resulting compounds into the active site of HIV-1 IN, to assess the potential affinity of the new compounds. The grid box for the docking calculation was centered on the centroid between the two Mg^2+^ ions which roughly represents the center of the active site. The cubic grid box was adjusted based on a size capable of accommodating ligands with a length 15 Å. As part of grid generation procedure, metal constraints for the receptor grids were also applied. The other options and parameters in this step were set as default and then docking of the library members into the homology modeled HIV-1 IN active site was performed using the extra precision method (XP) in CombiGlide docking. At the end of the process a focused combinatorial library of more than 37,000 compounds was obtained for each studied core-containing molecule. The total structures obtained from combinatorial screening were sorted on the basis of their GlideScore (Glide, Version 5.7, Schrödinger, LLC, New York, NY, 2011; Friesner et al., 2004). The compounds with the better XP Glide scores compared with the corresponding core-containing molecules were selected for further studies. The interactions of these compounds, into HIV-1 IN active site, were assessed by using ligand-interaction diagram implemented in Maestro suite and visualized by PyMOL (PyMOL Molecular Graphics System, Version 1.6-alpha, Schrödinger, LLC, New York, NY, 2013).

#### Molecular Properties Prediction

The molecules selected from *in silico* combinatorial screening were evaluated using a series of filtering criteria for drug-like properties. In this regard, QikProp application (QikProp, version 3.4, Schrödinger, LLC, New York, NY, 2011) implemented in the Maestro suite was used for ADME-T properties predictions (Rostami et al., 2015; Zaccagnini et al., 2017). This step was performed to select compounds from each library with appropriate physico-chemical properties using the range values recommended by QikProp. Especially, Lipinski's rules of five, membrane permeability, lipophilicity, cardiotoxicity, or potential interaction with *h*ERG K^+^ channel were considered as important criteria and investigated for filtering (Lipinski et al., 2001). Default settings were employed for these calculations. The compounds derived from the above-mentioned calculation were evaluated for their potential capability to behave as “Pan Assay Interference Compounds” (PAINS). This calculation was performed by means of FAFDrugs4.0 (http://fafdrugs4.mti.univ-paris-diderot.fr/) (Lagorce et al., 2008, 2011; Vallone et al., 2018; Brindisi et al., 2019). PAINS compounds are chemical compounds that tend to display activity against a wide range of targets by nonspecific interactions or by altering the results of the biological tests. The compounds containing this kind of moieties, that are often present in PAINS compounds, could be false positive hits and in general should be removed from the designed series (Baell and Holloway, 2010).

#### Quantum Polarized Ligand Docking (QPLD)

In order to narrow down the number of the potential INIs and for improving the reliability of the protocol, the quantum polarized ligand docking (QPLD) calculations were performed for the resulting compounds with satisfactory physico-chemical properties. These compounds were docked into the modeled HIV-1 IN using QPLD protocol implemented in Schrödinger 2011 (Schrödinger Suite 2011: QM-Polarized Ligand Docking protocol; Glide, Version 5.7, Schrödinger, LLC, New York, NY, 2011; Jaguar, version 7.8, Schrödinger LLC, New York, NY, 2011; QSite version 5.7, Schrödinger LLC, New York, NY, 2011) (Irwin et al., 2012). This step was added to improve the accuracy of classical docking calculation. In fact, this procedure aims to improve the partial charges on ligand atoms by replacing them with charges derived from quantum mechanical calculations on the ligand in the field of the receptor (Paolino et al., 2018). Within the QPLD framework, the ligand atoms are treated at the Quantum Mechanical (QM) level, whereas the IN enzyme including the Mg^2+^ ions as Molecular Mechanical (MM) region are described using the OPLS force field parameters. In this way, the same grid file previously employed in the CombiGlide step was used. The best docked compounds obtained from the previous CombiGlide docking calculations followed by the evaluation of the physico-chemical properties, were selected in the ligand option. In the first step of the QPLD calculation, compounds were initially docked into the active site of IN enzyme. The initial docking calculations were carried out using Glide standard precision (SP) docking protocol, generating 5 poses per docked molecule. In the second step, the polarizable ligand charges induced by the protein field were calculated with QSite software which is coupled with Jaguar quantum mechanics engine (Jaguar, version 7.8, Schrödinger LLC, New York, NY, 2011). In this regard, the QM charge calculations of the best scoring poses for each ligand were carried out using density functional theory (DFT) method with the B3LYP/6-31G^*^/LACVP^*^ basis set within the protein environment defined by the OPLS-2005 force field. Finally, the ligands with modified partial charges were redocked into the IN active site using Glide XP mode of docking considering 10 poses for each ligand. The potential inhibitors were selected based on the lower values of XP GlideScore and the key interactions between the ligand and HIV-1 IN active site.

#### Ligand Binding Energy Calculations

The best docked pose of ligands selected from previous QPLD calculations were subjected to a subsequent analysis with MM-GBSA process implemented in Prime software (Prime, version 3.0, Schrödinger LLC, New York, NY, 2011) (Brindisi et al., 2015). This method was employed to predict binding affinity and relative free-binding energy (ΔG_bind_) between ligands and HIV-1 IN with further accuracy. The MM-GBSA approach combines MM energies with a continuum solvent generalized Born (GB) model for polar solvation and with a solvent-accessible surface area (SASA) for non-polar solvation term. In this way, the best ligand poses were subjected to energy minimization by local optimization feature in the Prime. During this process, the ligand strain energy was also considered. Ligand binding energies were calculated using the OPLS-2005 force field and generalized-Born/surface area continuum solvent model as previously reported by us (Brindisi et al., 2015, 2016; Maquiaveli et al., 2016; Brogi et al., 2017a; Vallone et al., 2018).

#### Molecular Dynamics Simulation

MD simulations studies were performed by means of Desmond 4.8 academic version, provided by D. E. Shaw Research (“DESRES”), using Maestro as graphical interface (Desmond Molecular Dynamics System, version 4.8, D. E. Shaw Research, New York, NY, 2016. Maestro-Desmond Interoperability Tools, version 4.8, Schrödinger, New York, NY, 2016). The calculation was performed using the Compute Unified Device Architecture (CUDA) API (Nickolls et al., 2008) employing two NVIDIA GPU (Brindisi et al., 2019). The calculation was performed on a system comprising 72 Intel Xeon E5-2695 v4@2.10 GHz processors and two NVIDIA GeForce 1070 GTX GPU. The complexes **HPCAR-28**/IN, **HPCAR-89**/IN, and **HPCAR-142**/IN were prepared by Protein Preparation Wizard protocol. The complexes were positioned into an orthorhombic box filled with water (TIP3P model). OPLS_2005 force field was used in MD calculation. The physiological concentration of monovalent ions (0.15 M) was simulated by adding Na^+^ and Cl^−^ ions. Constant temperature (300 K) and pressure (1.01325 bar) were employed with NPT (constant number of particles, pressure, and temperature) as ensemble class. RESPA integrator (Humphreys et al., 1994) was used in order to integrate the equations of motion, with an inner time step of 2.0 fs for bonded interactions and nonbonded interactions within the short-range cut-off. Nose-Hoover thermostats (Hoover, 1985) were used to maintain the constant simulation temperature, and the Martyna-Tobias-Klein method (Martyna et al., 1994) was used to control the pressure. Long-range electrostatic interactions were evaluated adopting particle-mesh Ewald method (PME). The cut-off for van der Waals and short-range electrostatic interactions was set at 9.0 Å. The equilibration of the systems was performed with the default protocol provided in Desmond, which consists of a series of restrained minimizations and MD simulations used to slowly relax the system. By following this protocol, a single trajectory of 100 ns was obtained. We performed five independent MD runs for each mentioned complex with an aggregate simulation time of 0.5 μs to provide more reliable results. The trajectory files were investigated by simulation interaction diagram tools, simulation quality analysis and simulation event. The described applications were used to generate all plots regarding MD simulations analysis included in the manuscript as reported in the Results and Discussion section.

### Chemistry

All reactants and reagents were purchased from Alfa Aesar and Sigma–Aldrich as “synthesis grade.” Chemical reactions were monitored by analytical thin-layer chromatography (TLC) using several solvent systems with different polarity on Merck Silica Gel 60 F_254_ (0.040–0.063 mm) with detection by UV. Merck Silica Gel 60 (0.040–0.063 mm) was used for column chromatography. ^1^H NMR and ^13^C NMR spectra were recorded on a Varian 300 MHz (USA) spectrometer using the residual signal of the deuterated solvent as internal standard. Splitting patterns of signals are indicated as singlet (s), doublet (d), triplet (t), multiplet (m), broad (br), and doublet of doublet (dd). The values of chemical shifts (δ) are reported in ppm and coupling constants (*J*) in hertz (Hz). Electrospray ionization-mass spectrometric (ESI-MS) were acquired with an Agilent 1100 series LC/MSD spectrometer equipped with a multimode ion source and by using methanol as solvent.

#### 3-(Benzyloxy)-6-Methyl-4-oxo-4H-Pyran-2-Carboxylic Acid, BPCA

This key carboxylic acid intermediate was prepared according to a previously reported procedure (Sirous et al., 2019).

#### Procedures for the Synthesis of Amine Fragments AM (1–3)

With the aim of synthesizing the representative hit candidates, three kinds of different amine fragments were applied for amide coupling with the carboxylic acid functional group of intermediate **BPCA**. The required amine compounds were synthesized using the following methods.

#### Procedure for the Preparation of (3-Fluoro-5-(pyridin-2-yl)phenyl)methanamine (AM-1)

##### 3-Fluoro-5-(pyridin-2-yl)benzonitrile (3)

Starting from 2-bromopyridine **1**, a Suzuki coupling with (3-cyano-5-fluorophenyl)boronic acid **2** catalyzed by tetrakis (tri-phenylphosphine) palladium (0) provided the phenyl-pyridine derivative **3**. In this reaction tetrakis was generated *in situ* from palladium (II) acetate and triphenylphosphine (Tan et al., 2014). To a vigorously stirred yellowish solution of palladium (II) acetate (0.28 g, 1.26 mmol, 0.2 eq) and triphenylphosphine (1.66 g, 6.33 mmol, 1 eq) in 4 mL dioxane/water (3:1), a premixed solution of 2-bromopyridine **1** (1.00 g, 6.33 mmol, 1 eq) in dioxane (50 mL), and a solution of potassium carbonate (2.62 g, 19 mmol, 3 eq) in water (28 mL) was added. The reaction mixture was allowed to stir for 15 min under N_2_. This step was followed by the drop-wise addition of a solution of (3-cyano-5-fluorophenyl)boronic acid **2** (1.15 g, 6.96 mmol, 1.1 eq) in 8 mL dioxane/water (4:1) via a syringe. After the final addition, the reaction mixture was refluxed at 100°C in an oil bath under N_2_. The progression of the reaction was monitored by TLC. The reaction was completed after 24 h. After that, the reaction mixture was cooled down to the room temperature and subsequently filtered through a short Celite pad. The filter cake was washed with dichloromethane (25 mL). The filtrate solution was diluted with water and extracted with dichloromethane (3 × 100 mL). The organic layers were combined, dried over sodium sulfate, filtered and then evaporated *in vacuo* to yield a crude product. The crude product was purified by flash chromatography on silica gel, eluting with 9:1 petroleum ether: ethyl acetate, to give **3** as a white solid (Tan et al., 2014). Yield: 64%, ^1^H NMR (300 MHz, CDCl_3_) δ (ppm): 8.75 (1H, d, *J* = 6.25 Hz, C6′-H), 8.42 (1H, d, *J* = 6.00 Hz, C4-H), 7.80 (2H, s, C6-H, and C2-H), 7.62–7.72 (1H, m, C4′-H), 7.22–7.40 (2H, m, C3′-H and C5′-H).

##### (3-Fluoro-5-(pyridin-2-yl)phenyl)methanamine (AM-1)

Dry NiCl_2_ was prepared from hydrated NiCl_2_. In this way, NiCl_2_•6H_2_O was used after drying in an oven at 250°C until its color turned from green to golden yellow. Then, it was powdered and stored in a vacuum desiccator for reaction. In a typical procedure (Caddick et al., 2003), nitrile compound **3** (0.2 g, 1.00 mmol, 1 eq) and anhydrous nickel (II) chloride (0.13 g, 1 mmol, 1 eq) were dissolved in dry ethanol (8 mL). Then, sodium borohydride (0.11 g, 3 mmol, 3 eq) was cautiously added in three portions to the vigorously stirred reaction mixture at room temperature. A black precipitate appeared during the addition of NaBH_4_. When the addition of NaBH_4_ was completed, stirring was continued and the progress of the reaction was monitored by TLC. After the complete disappearance of the nitrile compound in almost 15 min, the reaction mixture was filtered through a Celite pad. The filtered nickel boride precipitate was washed with ethanol (10 mL). The filtrate was collected, diluted with water (30 mL), and extracted with ethyl acetate (3 × 30 mL). The organic phase was combined, dried over sodium sulfate, filtered and concentrated on a rotary vacuum evaporator to afford a crude product. The crude product was purified by flash chromatography on silica gel, eluting with 4:1 chloroform:methanol to give the corresponding amine compound **AM-1** as a white solid (Caddick et al., 2003). Yield: 65%, ^1^H NMR (300 MHz, DMSO-d_6_) δ (ppm): 8.66 (2H, s, NH_2_), 8.12 (1H, d, *J* = 6Hz, C6′-H), 7.69–7.98 (3H, m, Ar), 7.59–7.70 (1H, br, C4′-H), 7.20–7.40 (2H, m, C3′-H and C5′-H), 4.76 (2H, s, C*H*_2_NH_2_).

#### Procedure for the Preparation of (3-(2-methyl-1H-imidazol-4-yl)phenyl)methanamine (AM-2)

##### 4,5-Dibromo-2-methyl-1H-imidazole (5)

2-Methyl-1*H*-imidazole **4** (10 g, 0.122 mol) was dissolved in 300 mL chloroform and cooled to the temperature between 0 and −5°C using salty ice bath. 48.66 g (15.60 mL, 0.305 mol) bromine was added to the reaction mixture drop-wise via a dropping funnel over 20 min. The reaction mixture was allowed to stir at room temperature. The progression of the reaction was monitored by TLC. The reaction was completed after 20 h. During this time, the reaction product precipitated as an orange solid. In the next step, the reaction mixture was cooled to 0°C in a salty ice bath and 250 mL of NaOH (2 N) was added drop-wise to the reaction mixture in order to quench unreacted bromine. The orange precipitate was filtered off and washed with water, dried *in vacuo* at 40°C for 12 h to give the yellow solid of **5** (Alonso-Alija et al., 2003). Yield: 43%, ^1^H NMR (300 MHz, DMSO-d_6_) δ (ppm): 8.68 (1H, brs, NH), 2.39 (3H, s, CH_3_).

##### 4-Bromo-2-methyl-1H-imidazole (6)

4,5-Dibromo-2-methyl-1*H*-imidazole **5** (5.0 g, 20.84 mmol) was suspended with sodium sulfite (80 g, 635 mmol) in 200 mL water and 100 mL ethanol. The suspension was refluxed at 100°C in an oil bath. The progression of the reaction was monitored by TLC. The reaction was completed after 48 h and the reaction mixture was extracted with ethyl acetate (3 × 100 mL). The organic phases were collected, dried over sodium sulfate, filtered and then evaporated *in vacuo* to yield a white solid as pure product **6** (Alonso-Alija et al., 2003). Yield: 75%, ^1^H NMR (300 MHz, DMSO-d_6_) δ (ppm): 12.00 (1H, brs, NH), 7.05 (1H, d, *J* = 1.80 Hz, C5-H), 2.21 (3H, s, CH_3_).

##### 3-(2-Methyl-1H-imidazol-4-yl)benzonitrile (8)

Nitrile compound **8** was synthesized according to same Suzuki procedure described for the preparation of compound **3** (Tan et al., 2014). Briefly, starting from a solution of palladium (II) acetate (0.42 g, 1.86 mmol, 0.2 eq) and triphenylphosphine (2.44 g, 9.32 mmol, 1 eq) in 6 mL dioxane/water (5:1), a solution of bromo-imidazole derivative **6** (1.5 g, 9.32 mmol, 1 eq) in dioxane (70 mL), a solution of potassium carbonate (3.87 g, 28 mmol, 3 eq) in water (40 mL) and a solution of (3-cyanophenyl) boronic acid **7** (1.51 g, 10.252 mmol, 1.1 eq) in 10 mL dioxane/water (5:1) were used in Sequence. Crude product was purified by flash chromatography on silica gel using 3:1 petroleum ether: ethyl acetate as the eluent to yield **8** as a white solid (Tan et al., 2014). Yield: 70%, ^1^H NMR (300 MHz, DMSO-d_6_) δ (ppm): 11.95 (1H, brs, NH), 8.10 (1H, s, C2′-H), 8.00 (1H, d, *J* = 9.40 Hz, C6′-H), 7.45–7.68 (3H, m, C5-H, C4′-H, C5′-H), 2.20 (3H, s, CH_3_). ESI-MS (+) m/z (%): 183.9 [M+H]^+^ (100).

##### (3-(2-Methyl-1H-imidazol-4-yl)phenyl)methanamine (AM-2)

Amine compound **AM-2** was synthesized according to the same procedure described for reduction of nitrile **3** to the corresponding primary amine **AM-1** (Caddick et al., 2003). In this way, starting from nitrile compound **8** (0.5 g, 2.73 mmol, 1 eq), 0.353 gr (2.73 mmol, 1 eq) of anhydrous nickel (II) chloride, and 0.31 g (8.19 mmol, 3 eq) sodium borohydride were used. The purification was performed via silica flash column chromatography using 4:1 chloroform: methanol as the eluent to yield the corresponding amine product **AM-2** as a white solid (Caddick et al., 2003). Yield: 61%, ^1^H NMR (300 MHz, DMSO-d_6_) δ (ppm): 11.72 (1H, brs, NH), 7.70–8.50 (5H, m, C5-H, C2′-H, C4′-H, C5′-H and C6′-H), 3.70 (2H, s, CH_2_NH_2_), 2.25 (3H, s, CH_3_).

#### Procedure for the Preparation of (3-(1H-pyrrol-1-yl)phenyl)methanamine (AM-3)

##### 3-(1H-Pyrrol-1-yl)benzonitrile (11)

Phenylpyrrole **11** was synthesized through a Clauson–Kaas reaction with 3-aminobenzonitrile **9** (Chatzopoulou et al., 2013). To a solution of 3-aminobenzonitrile **9** (1.00 g, 8.46 mmol, 1 eq) in 15.0 mL of 1,4-dioxane, 2,5-dimethoxytetrahydrofuran **10** (1.23 g, 9.306 mmol, 1.1 eq) dissolved in 7.0 mL of 1,4-dioxane were added. The reaction mixture was refluxed for 5 min, and then 3 mL of hydrochloric acid 5 N was added drop-wise. The reaction mixture was refluxed for 25 min until the reaction was completed. After cooling to room temperature, water was added to the reaction mixture. The reaction mixture was extracted with dichloromethane (3 × 50 mL). The organic layer was dried over anhydrous sodium sulfate, filtered, and concentrated *in vacuo*. Flash chromatography on silica gel, eluting with 4:1 petroleum ether: ethyl acetate, afforded compound **11** as an amorphous white solid (Chatzopoulou et al., 2013). Yield: 82%, ^1^H NMR (300 MHz, CDCl_3_) δ (ppm): 7.48–7.52 (4H, m, Ar), 7.08 (2H, t, *J* = 3.00 Hz, C2′-H, C5′-H), 6.79 (2H, t, *J* = 3.00 Hz, C3′-H, C4′-H).

##### (3-(1H-Pyrrol-1-yl)phenyl)methanamine (AM-3)

Amine compound **AM-3** was synthesized according to the same procedure described for the reduction of nitrile **3** to the corresponding primary amine **AM-1** (Caddick et al., 2003). In this way, starting from nitrile **11** (0.5 g, 2.97 mmol, 1 eq), 0.385 g (2.97 mmol, 1 eq) of anhydrous nickel (II) chloride, and 0.337 g (8.91 mmol, 3 eq) sodium borohydride were used. The purification was performed via silica column chromatography using 4:1 chloroform: methanol as the eluent to yield the corresponding amine **AM-3** as a white solid (Caddick et al., 2003). Yield: 75%, ^1^H NMR (300 MHz, DMSO-d_6_) δ (ppm): 7.52 (1H, s, C_2_-H), 7.27–7.42 (4H, m, 3H: C3-H, C4-H and C5-H; 1H: C2′-H), 7.18 (1H, d, *J* = 4.40 Hz, C5′-H), 6.24 (2H, s, C3′-H, C4′-H), 3.77 (2H, s, C*H*_2_NH_2_).

#### General Procedure for the Synthesis of 3-(benzyloxy)-6-methyl-N-(Substituted benzyl)-4-oxo-4H-pyran-2-carboxamide Derivatives, BPCAR

To a vigorously stirred suspension of intermediate **BPCA** (100 mg, 0.38 mmol, 1 eq) in dry dichloromethane (8 mL), 1-ethyl-3-(3-dimethylaminopropyl) carbodiimide hydrochloride (EDCI) (72.5 mg, 0.38 mmol, 1 eq) was added and the mixture was stirred for 30 min under N_2_ to provide a clear yellow solution. Then, *N*-hydroxysuccinimide (NHS) (43.7 mg, 0.38 mmol, 1 eq) was added to the stirring solution and the mixture was allowed to stir for 3 h under N_2_ to produce the activated ester **12** as reported in the Chemistry details in the Result and Discussion section. After this step and complete consumption of the starting material **BPCA**, desired prepared amine fragment (1.5 eq) was added to the reaction mixture. The reaction was stirred at room temperature for 3 days under N_2_. During this time, the progression of the reaction was monitored by TLC. Then, the reaction mixture was poured into a separatory funnel and the dichloromethane layer was washed with water (2 × 20 mL). The organic phase was collected, dried over anhydrous sodium sulfate, filtered, and concentrated *in vacuo* to yield a crude solid. The obtained solid was purified via silica flash column chromatography to give the desired carboxamide product as a pure substance (Sheehan et al., 1965; Sirous et al., 2019).

#### 3-(Benzyloxy)-N-(3-fluoro-5-(pyridin-2-yl)benzyl)-6-methyl-4-oxo-4H-pyran-2-carboxamide (BPCAR-28)

Carboxamide derivative **BPCAR-28** was prepared according to the general procedure, using amine compound **AM-1** (115 mg, 0.57 mmol, 1.5 eq). The purification of the crude product using silica flash column chromatography, eluting with 1:1 petroleum ether: ethyl acetate solution, afforded the carboxamide product **BPCAR-28** as a white solid. Yield: 44%, ^1^H NMR (300 MHz, CDCl_3_) δ (ppm): 8.74 (1H, d, *J* = 5.5 Hz, C6′-H), 8.18(1H, brs, NHCH_2_), 7.88 (1H, d, *J* = 5.5 Hz, Ar: C4-H), 7.76 (2H, s, Ar: C2-H and C6-H), 7.18–7.40 (6H, m, 5H: OCH_2_C_6_*H*_5_; 1H: C4′-H), 7.02–7.16 (2H, m, C3′-H and C5′-H), 6.24 (1H, s, C5-H), 5.36 (2H, s, OC*H*_2_-C_6_H_5_), 4.44 (2H, d, *J* = 8.00 Hz, NHC*H*_2_-C_6_H_4_), 2.38 (3H, s, 6-CH_3_).

#### 3-(Benzyloxy)-6-methyl-N-(3-(2-methyl-1H-imidazol-4-yl)benzyl)-4-oxo-4H-pyran-2-carboxamide (BPCAR-89)

Carboxamide derivative **BPCAR-89** was prepared according to the general procedure, using amine compound **AM-2** (0.107 g, 0.57 mmol, 1.5 eq). The purification of the crude product using silica flash column chromatography, eluting with 1:1 petroleum ether: ethyl acetate solution, afforded the carboxamide product **BPCAR-89** as a white solid. Yield: 38%, ^1^H NMR (300 MHz, Acetone-d_6_) δ (ppm): 8.41 (1H, brs, NHCH_2_), 7.82 (1H, s, C2′-H), 7.70 (1H, d, *J* = 12.00 Hz, C4′-H),7.23–7.40 (7H, m, 5H:OCH_2_-C_6_*H*_5_; 2H: C5′-H and C6′-H), 7.18 (1H, d, *J* = 12.00 Hz, C5″-H), 6.28 (1H, s, C5-H), 5.30 (2H, s, OC*H*_2_-C_6_H_5_), 4.50 (2H, d, *J* = 6.00 Hz, NHC*H*_2_-C_6_H_4_), 2.40 (3H, s, CH_3_), 2.24 (3H, s, CH_3_).

#### N-(3-(1H-Pyrrol-1-yl)benzyl)-3-(benzyloxy)-6-methyl-4-oxo-4H-pyran-2-carboxamide (BPCAR-142)

Carboxamide derivative **BPCAR-142** was prepared according to the general procedure, using amine compound **AM-3** (98.2 mg, 0.57 mmol, 1.5 eq). The purification of the crude product using silica flash column chromatography, eluting with 1:1 petroleum ether: ethyl acetate solution, afforded the carboxamide product **BPCAR-142** as a white solid. Yield: 48%, ^1^H NMR (300 MHz, CDCl_3_) δ (ppm): 8.12 (1H, t, *J* = 8.00 Hz, NHCH_2_), 7.16–7.38 (8H, m, 5H: OCH_2_C_6_H_5_; 3H: C2′-H, C4′-H and C5′-H), 7.01–7.08 (3H, m, 2H: C2″-H, C5″-H; 1H: C6′-H), 6.36 (2H, t, *J* = 3.00 Hz, C3″-H, C4″-H), 6.25 (1H, S, C5-H), 5.32 (2H, s, OC*H*_2_C_6_H_5_), 4.46 (2H, d, *J* = 8.30 Hz, NHC*H*_2_C_6_H_4_), 2.35 (3H, s, 6-CH_3_).

#### General Procedure for the Synthesis of 3-hydroxy-6-methyl-N-(substituted benzyl)-4-oxo-4H-pyran-2-carboxamide derivatives, HPCAR

40 mg of each of the desired BPCAR derivatives was dissolved in dry dichloromethane (3 mL) and flushed with nitrogen. Then, the reaction mixture was cooled to the temperature between 0 and −5°C in salty ice bath and the 1 M solution of boron tribromide in dichloromethane (3 eq) was slowly added drop-wise via a syringe. The reaction mixture was allowed to stir at room temperature and the reaction progress was monitored by TLC. The reaction was completed after almost 3 h. The excess BBr_3_ was eliminated at the end of the reaction by the addition of cold methanol (5 mL) to the reaction mixture at 0°C and left to stir for half an hour. The mixture was concentrated to dryness in vacuum and the residue was dissolved several times in methanol and evaporated. This residue was purified by flash column chromatography to afford the final pure product (Ma and Hider, 2015; Sirous et al., 2019).

#### N-(3-Fluoro-5-(pyridin-2-yl)benzyl)-3-hydroxy-6-methyl-4-oxo-4H-pyran-2-carboxamide (HPCAR-28)

Compound **HPCAR-28** was prepared according to the general debenzylation procedure, starting from compound **BPCAR-28** (40 mg, 0.09 mmol, 1 eq) and 1 M solution of boron tribromide in CH_2_Cl_2_ (46 μL, 0.27 mmol, 3 eq). Purification using flash column chromatography (eluent: dichloromethane: methanol; 80:20 v/v) afforded a white solid as final product **HPCAR-28**. Yield: 71%, ^1^H NMR (300 MHz, DMSO-d_6_) δ (ppm): 8.60 (1H, d, *J* = 5.5 Hz, C6′-H), 8.12 (1H, brs, NHCH_2_), 7.74 (1H, d, *J* = 5.5 Hz, Ar: C4-H), 7.62 (2H, s, Ar: C2-H and C6-H), 7.12–7.34 (1H, br, C4′-H), 7.00–7.13 (2H, m, C3′-H and C5′-H), 6.22 (1H, s, C5-H), 4.34 (2H, d, *J* = 8.00 Hz, NHC*H*_2_-C_6_H_4_), 2.34 (3H, s, 6-CH_3_). ^13^C NMR (DMSO-d6) δ (ppm): 173.55 (4-C = O), 164.84 (CONH), 162.72 (C-3), 162.48 (Ar: C_3′_-F, d, ^1^*J*_C−F_: 220.4 Hz), 152.15 (Ar), 147.39 (Ar), 144.05 (C-2), 143.86 (Ar), 136.14 (C-6), 132.32 (Ar), 132.12 (Ar), 124.00 (Ar), 123.09 (Ar), 122.28 (Ar), 116.60 (Ar), 114.11 (Ar), 108.63 (C-5), 42.04 (NHCH_2_), 19.31 (6-CH_3_). ESI-MS (+) m/z (%): 355.3 [M+H]^+^ (100).

#### 3-Hydroxy-6-methyl-N-(3-(2-methyl-1H-imidazol-4-yl)benzyl)-4-oxo-4H-pyran-2 carboxamide (HPCAR-89)

Compound **HPCAR-89** was prepared according to the general procedure described above, starting from compound **BPCAR-89** (40 mg, 0.09 mmol, 1 eq) and 1 M solution of boron tribromide in CH_2_Cl_2_ (46 μL, 0.27 mmol, 3 eq). Purification using flash column chromatography (eluent: dichloromethane: methanol; 80:20 v/v) afforded a white solid as final product **HPCAR-89**. Yield: 82%, ^1^H NMR (300 MHz, DMSO-d_6_) δ (ppm): 11.80 (1H, brs, NH of imidazole ring), 10.63 (1H, brs, NHCH_2_),7.60 (1H, s, C2′-H), 7.58 (1H, d, *J* = 12.00 Hz, C4′-H), 7.38 (1H, s, C5"-H), 7.20 (1H, t, *J* = 12.00 Hz, C5′-H), 7.05 (1H, d, *J* = 12.00 Hz, C6′-H), 6.20 (1H, s, C5-H), 4.40 (2H, d, *J* = 6.00 Hz, NHC*H*_2_-C_6_H_4_), 2.24 (6H, s, CH_3_-a and CH_3_-b). ^13^C NMR (DMSO-d6) δ (ppm): 173.53 (4-C = O), 164.78 (CONH), 162.72 (C-3), 156.60 (C_3_H_4_N_2_: C2), 151.02 (C_3_H_4_N_2_: C5), 147.44 (C-2), 142.54 (Ar), 136.14 (C-6), 134.64 (Ar), 129.47 (Ar), 128.30 (Ar), 125.52 (Ar), 123.14 (C_3_H_4_N_2_: C4), 117.21 (Ar), 112.65 (C-5), 41.53 (NHCH_2_), 19.30 (6-CH_3_), 16.60 (CH_3_-C_3_H_4_N_2_). ESI-MS (+) m/z (%): 340.1 [M+H]^+^ (100).

#### N-(3-(1H-Pyrrol-1-yl)benzyl)-3-hydroxy-6-methyl-4-oxo-4H-pyran-2-carboxamide (HPCAR-142)

Compound **HPCAR-142** was prepared according to the general procedure, starting from compound **BPCAR-142** (40 mg, 0.096 mmol, 1 eq) and 1 M solution of boron tribromide in CH_2_Cl_2_ (49 μL, 0.288 mmol, 3 eq). Purification using flash column chromatography (eluent: dichloromethane: methanol; 80:20 v/v) afforded a white solid as final product **HPCAR-142**. Yield: 86%, ^1^H NMR (300 MHz, DMSO-d_6_) δ (ppm): 10.30 (1H, brs, NHCH_2_), 7.2–7.56 (4H, m, C2′-H, C4′-H, C5′-H, C6′-H), 7.0–7.20 (2H, m, C2″-H, C5″-H), 6.23 (2H, d, *J* = 7.50 Hz, C3″-H, C4″-H), 6.18 (1H, s, C5-H), 4.50 (2H, d, *J* = 7.00 Hz, NHC*H*_2_), 2.30 (3H, s, 6-CH_3_). ^13^C NMR (DMSO-d6) δ (ppm): 173.55 (4-C = O), 164.79 (CONH), 162.72 (C-3), 147.51 (C-2), 142.52 (Ar), 136.19 (C-6), 135.31 (Ar), 128.90 (Ar), 124.37 (Ar), 120.05 (C_4_H_4_N: C2, C5), 119.30 (Ar), 117.43 (Ar), 112.67 (C-5), 108.77 (C_4_H_4_N: C3, C4), 41.96 (NHCH_2_), 20.65 (6-CH_3_). ESI-MS (+) m/z (%): 324.9 [M+H]^+^ (30), 346.7 [M+Na]^+^ (100).

### Biological Evaluation

#### Integrase Assays

The enzymatic integration reactions were carried out as previously described with minor modifications (Debyser et al., 2001; Christ et al., 2011). To determine the susceptibility of the HIV-1 IN enzyme to different compounds, an enzyme-linked immunosorbent assay (ELISA) adapted from Hwang et al. was used (Hwang et al., 2000). The overall integration assay uses an oligonucleotide substrate for which one oligonucleotide (5′-ACTGCTAGAGATTTTCCACACTGACTAAAAGGGTC-3′) is labeled with biotin at the 3′ end and the other oligonucleotide (5′-GACCCTTTTAGTCAGTGTGGAAAATCTCTAGCAGT-3′) is labeled with digoxigenin at the 5′ end. For the strand transfer assay, a pre-cleaved oligonucleotide substrate (the second oligonucleotide lacks GT [underlined] at the 3′ end) was used. The IN enzyme was diluted in 750 mM NaCl, 10 mM Tris (pH: 7.6), 10% glycerol, and 1 mM β-mercaptoethanol. To perform the reaction, 4 μL of diluted IN (corresponding to a concentration of 1.6 μM) and 4 μL of annealed oligonucleotides (7 nM) were added in a final reaction volume of 40 μL containing 10 mM MgCl_2_, 5 mM dithiothreitol, 20 mM HEPES (pH 7.5), 5% polyethylene glycol, and 15% dimethyl sulfoxide. As such, the final concentration of IN in this assay was 160 nM. The reaction was carried out for 1 h at 37°C. Reaction products were denatured with 30 mM NaOH and detected by ELISA on avidin-coated plates. For determining the effect of compounds on the 3′-processing activity a classical cleavage assay with detection of products by denaturing gel electrophoresis was performed as described previously (Debyser et al., 2001; Christ et al., 2011). Briefly, 0.2 pmol of the radioactive labeled oligonucleotide substrate (INT1, ^32^P-5′ TGTGGAAAATCTCTAGCAGT3′; INT2, 5′ACTGCTAGAGATTTTCCACA 3′) and 10 nmol IN in a final volume of 10 μL was incubated for 1 h at 37°C. The final reaction mixture contained 20 mM HEPES (pH 7.5), 5 mM dithiothreitol (DTT), 10 mM MgCl_2_, 0.5% (v/v) polyethylene glycol 8000, 15% DMSO. IN was diluted previously in 750 mM NaCl, 10 mM Tris (pH 7.6), 10% glycerol and 1 mM β-mercaptoethanol. The reactions were stopped by the addition of formamide loading buffer (95% formamide, 0.1% xylene cyanol, 0.1% xylene cyanol, 0.1% bromophenol blue, and 0.1% sodium dodecyl sulfate). Samples were loaded on a 15% denaturing polyacrylamide/ureum gel. The extent of 3′-processing or DNA strand transfer was based on measuring the respective amounts of −2 bands or strand transfer products relative to the intensity of the total radioactivity present in the lane. These data were determined using the OptiQuant Acquisition and Analysis software (Perkin Elmer Corporate, Fremont, CA).

#### *In vitro* Anti-HIV and Drug Susceptibility Assays

The inhibitory effect of antiviral drugs on the HIV-induced cytopathic effect (CPE) in human lymphocyte MT-4 cell culture was determined by the MT-4/MTT-assay (Pauwels et al., 1988). This assay is based on the reduction of the yellow colored 3-(4,5-dimethylthiazol-2-yl)-2,5-diphenyltetrazolium bromide (MTT) by mitochondrial dehydrogenase of metabolically active cells to a blue formazan derivative, which can be measured spectrophotometrically. The 50% cell culture infective dose of the HIV strains was determined by titration of the virus stock using MT-4 cells. For the drug susceptibility assays, MT-4 cells were infected with 100–300 50% cell culture infective doses of the HIV strains in the presence of 5-fold serial dilutions of the antiviral drugs. The concentration of the compound achieving 50% protection against the CPE of HIV, which is defined as the 50% effective concentration (EC_50_), was determined. In parallel, the concentration of the compound destroying 50% of the MT-4 cells, which is defined as the 50% cytotoxic concentration (CC_50_), was determined as well.

## Results and Discussion

The main purpose of the present study is to identify novel chemical entities derived from HPCARs scaffold as new and useful hit compounds as HIV-1 INIs. Accordingly, an integrated computational protocol based on combinatorial library design protocol, physico-chemical properties prediction, molecular docking calculations, and MD simulation was developed in a stepwise filtering approach (Figure 3). The identified hit compounds were synthesized and submitted to biological evaluation in order to validate the proposed *in silico* strategy.

### Generation of Combinatorial Hits Using CombiGlide

As the first step of the developed *in silico* protocol, HPCAR derivatives with *n* = 1 or 2 (Figures 2, 3) were submitted to CombiGlide software as a combinatorial docking tool. In each case, combinatorial virtual screening was applied in order to replace the aromatic groups of the original core, applying side-chain hopping method. The prepared sets of amine fragments, available in the library of reagents, were used to replace the original substituents at each defined attachment point (Figure 3). Variation in aromatic group resulted in the generation of a combinatorial library of more than 37,000 hit compounds for each studied core-containing molecule. The compounds from each new combinatorial library were sorted by GlideScore values. Only derivatives with score values lower than −6.0 kcal/mol were considered. The selected molecules were further analyzed by visual inspection to find compounds with an appropriate binding mode according to the key interactions found for HIV-1 INIs. From this first filter, 1,803 combinatorial compounds were chosen for the next step.

### Molecular Properties Prediction

One of the major goals in drug discovery is the identification of innovative small molecular scaffolds exhibiting high efficacy and selectivity against the desired target along with a satisfactory ADME-T profile. Thus, the second filter in the screening workflow consisted in the prediction of the ADME-T properties and drug-like behavior of the above-mentioned 1,803 compounds using QikProp software. This step was performed to select molecules possessing satisfactory predicted membrane permeability (QPPCaco-2 and QPPMDCK models > 100), appropriate lipophilicity (QPlogP) including capability to cross the blood brain barrier and drug-likeness properties in accordance with Lipinski's rule of five. The potential interaction with *h*ERG K^+^ channel (QPlog-HERG) was another key parameter considered in this step of filtering. 146 out of 1,803 compounds were predicted to have pharmacokinetic properties in the appropriate range. Moreover, the resulting compounds were filtered for behaving as PAINS using FAF-Drugs4 tool. Among 146 compounds, only two molecules contain sub-structural features that marked them as “frequent hitters” in high throughput screens. Finally, 144 candidates passed this step of screening and were chosen for the next step. A list of these top candidates with improved ADMET properties was provided in the Table S1.

### Quantum Polarized Ligand Docking Simulation

The resulting 144 potential hit molecules were further computationally analyzed using QPLD calculations for guaranteeing a better prediction of their binding mode into HIV-1 IN active site. This docking protocol could provide a more accurate treatment of electronic interactions especially within metalloproteins active site, leading to the improvement of the accuracy of the docking results (Cho et al., 2005; Illingworth et al., 2008; Paolino et al., 2018). In this step, the potential inhibitors were selected based on their lower XP GlideScore and on the ability to engage in critical interactions in the HIV-1 IN active site. At the end, a total of 76 hit candidates with the favorable XP GlideScore values were identified (Table S2). As reported in Table S2, the 76 selected hit molecules showed XP GlideScore values < −6 kcal/mol (the values of the cut-off filters for the *in silico* studies were chosen taking into consideration the values found for the reference compounds **RLT**, **EVG**, and **DTG**). The detailed analysis of QPLD results indicated that these compounds adopt a reasonable interfacial binding mode similar to that found for the approved HIV-1 INIs, namely **RLT**, **EVG**, and **DTG** (Rostami et al., 2015; Sirous et al., 2015, 2019). Consistent with docking models of HP derivatives previously reported (Sirous et al., 2019), the same interaction pattern was found for the best docked pose of all the selected hit molecules within HIV-1 IN active site. In this context, combinatorial hits perfectly occupied the DNA/IN interface with donor oxygen triad of MBG interacting with both Mg^2+^ ions through a bis-bidentate mode of chelation. This orientation enables the aromatic side chain of the molecule to sit in a hydrophobic pocket close to the active site generated by the displacement of the terminal adenosine on the 3′-end of the viral DNA. As a result, the terminal aromatic moiety of ligands participates in π-π stacking interactions with the viral DNA nucleosides, DC_16_ and DG_4_, and favorable hydrophobic contacts with the amino acids residues of the catalytic loop, Pro_145_, Gln_146_, and Gly_149_. Particularly, Pro_145_ and Gln_146_ are directly involved in separation of the viral DNA strands upon the ST reaction. This can reduce the catalytic loop mobility and thus physically hamper the binding of the host DNA (Dirac and Kjems, 2001; Dolan et al., 2009). In some cases, further stabilization of the ligand in the active site was mediated by H-bonds with Asn_117_, Pro_145_, Gln_146_, and Glu_152_ as well as nucleoside residues DG_4_, DC_16_, and DA_17_. For example, **HPCAR-40** was involved in hydrogen bond interactions with Asn_117_ and Glu_152_ and **HPCAR-144** formed hydrogen bonds with Gln_146_ and Glu_152_. Furthermore, in most of the docking models, the position of 4-pyran core of ligands was suitably located to establish strong hydrophobic interactions such as a π-π stacking with 3′-deoxyadenosine A_17_ (Table S2).

### Prioritization of Hit Compounds Based on Relative Ligand Binding Energy

Although it is well established that docking calculations are highly successful in offering reliable ligand poses within the protein binding site, they often fail to rank compounds with respect to their binding affinities. This poor correlation may be due to severe approximations and simplifications employed by scoring functions of various docking tools. The scoring functions like GlideScore do not consider some essential thermodynamics factors in the ligand binding energy calculations such as protein and ligand solvation energy terms (Pearlman and Charifson, 2001; Taylor et al., 2002). Thus in the subsequent step of our computational workflow, relative ligand binding energy calculations using MM-GBSA rescoring method were carried out on the best docked pose of the ligands obtained from the previously described docking simulation. This approach may offer more reliable measuring criteria to prioritize screened HIV-1 INIs hits for chemical synthesis and biological evaluations as HIV-1 INIs (Huang et al., 2006). Rescoring using MM-GBSA leads to minor changes of the ligand conformations within receptor site. These changes result from minimization of the ligand in receptor's environment and consequent stabilization of receptor-ligand complex. The estimated binding energy values < −25 kcal/mol were considered to retrieve final set of combinatorial hits. Final ranking of the ligands in this step of screening workflow resulted in the identification of 40 top hit compounds as novel HIV-1 INIs, possessing relevant binding affinities for HIV-1 IN active site. The structures of these compounds are shown in Table 2. The calculated ΔG_bind_ of the final selected hits along with their contributions to total binding energy from various energy components are provided in Table S3.

**Table 2:** Chemical structures of 40 top combinatorial hits identified at the end of computational screening workflow applied in the present study.

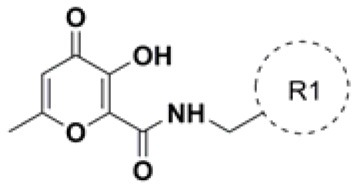					
**Cmpd**	**R**_**1**_	**Cmpd**	**R**_**1**_	**Cmpd**	**R**_**1**_
**HPCAR-1**	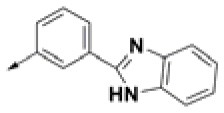	**HPCAR-33**	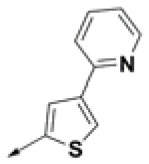	**HPCAR-89**	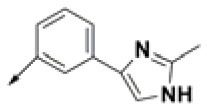
**HPCAR-2**	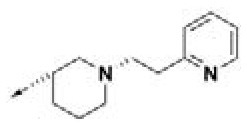	**HPCAR-35**	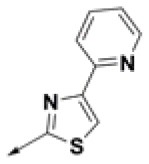	**HPCAR-90**	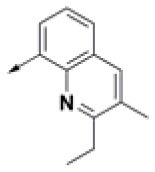
**HPCAR-6**	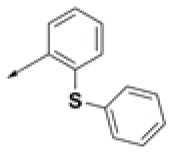	**HPCAR-37**	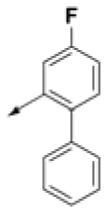	**HPCAR-91**	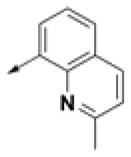
**HPCAR-7**	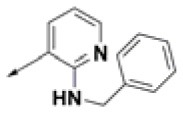	**HPCAR-41**	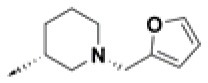	**HPCAR-92**	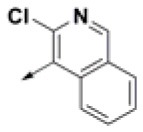
**HPCAR-8**	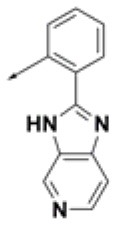	**HPCAR-44**	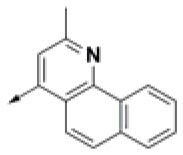	**HPCAR-108**	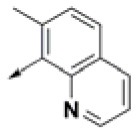
**HPCAR-14**	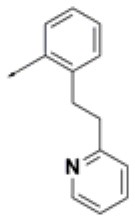	**HPCAR-45**	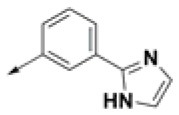	**HPCAR-111**	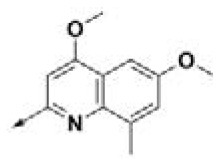
**HPCAR-15**	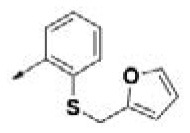	**HPCAR-46**	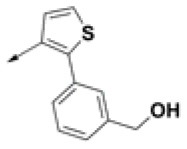	**HPCAR-114**	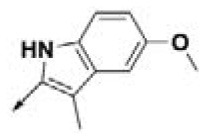
**HPCAR-22**	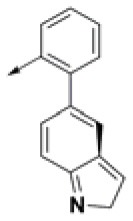	**HPCAR-52**	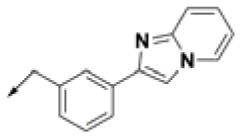	**HPCAR-123**	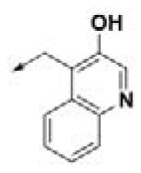
**HPCAR-23**	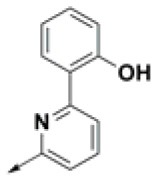	**HPCAR-54**	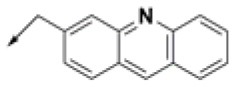	**HPCAR-126**	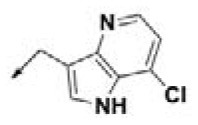
**HPCAR-25**	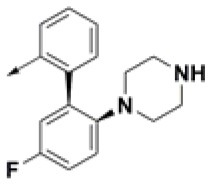	**HPCAR-55**	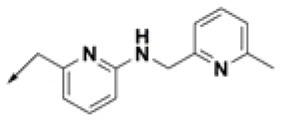	**HPCAR-130**	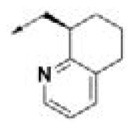
**HPCAR-26**	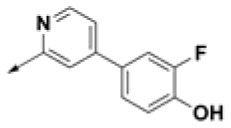	**HPCAR-56**	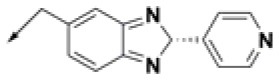	**HPCAR-140**	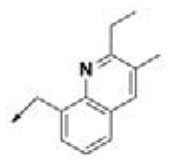
**HPCAR-28**	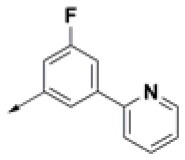	**HPCAR-66**	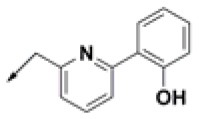	**HPCAR-142**	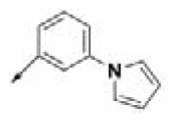
**HPCAR-29**	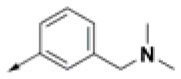	**HPCAR-69**	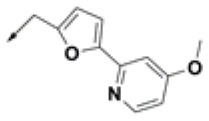	**HPCAR-144**	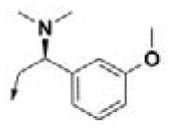
**HPCAR-30**	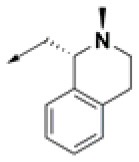				

*Three hit compounds (**HPCAR-28**, **HPCAR-89** and **HPCAR-142**) were selected from this final set for synthesis and biological assessment*.

Since the screened hit molecules share the same MBG, the main differences in ligand binding energies values between these inhibitors could be directly attributed to the hydrophobic aromatic moieties characterized by significant chemical diversity, including bicyclic and tricyclic structures. Inspection of energy terms in Table S3 revealed that all selected ligands showed high values of van der Waals interaction energy (ΔG_bindVdW_ values), contributing to the ligand binding energy which emphasizes critical importance of hydrophobic interactions in the stability of the ligand–protein complexes.

### Validation of Computational Screening Workflow

**Figure 4:**
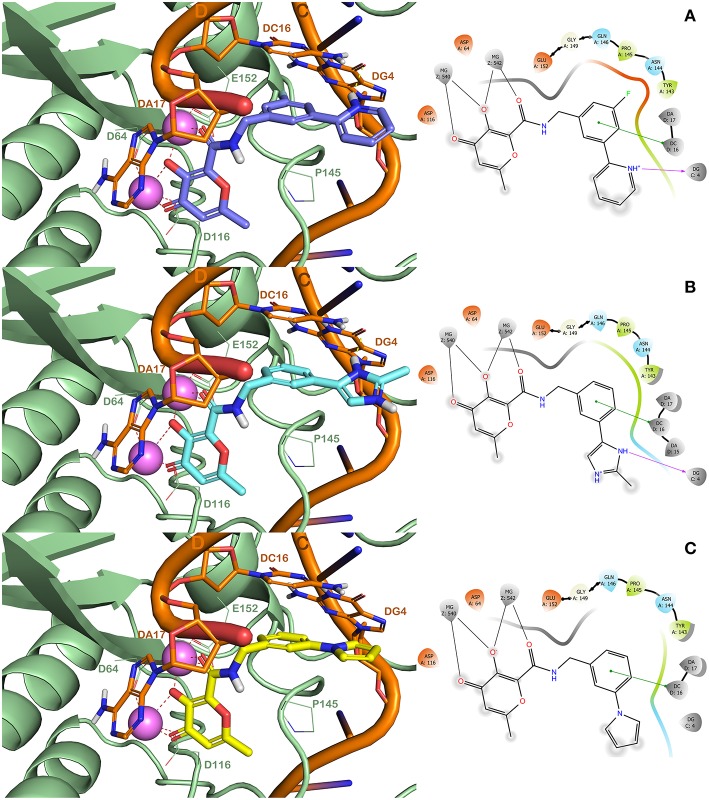
(Left) Binding modes of **HPCAR-28** (purple sticks, **A**), **HPCAR-89** (cyan sticks, **B**), and **HPCAR-142** (yellow sticks, **C**) in the active site of the modeled HIV-1 IN, as found by QPLD method. The solid ribbon model shows the backbone of the modeled HIV-IN, and the key amino acids of the binding site are shown as lines. Two Mg^2+^ ions are displayed as violet spheres, while the viral DNA strands are depicted in orange. Hydrogen and metal coordination bonds are represented by dashed lines. These figures were prepared using PyMOL. (Right) Ligand interaction diagrams for the selected compounds. These figures were prepared using Maestro.

In order to validate the developed computational protocol, three compounds (**HPCAR-28**, **HPCAR-89**, and **HPCAR-142** in Table 2) from final set of 40 hit candidates were selected and synthesized. The selection was performed considering the favorable computational scores, the binding modes, the structural differences and synthetic accessibility. The best docked poses along with the detailed interaction into the HIV-1 IN active site of these representative compounds are depicted in Figure 4.

The compounds were able to establish a bis-bidentate chelation of the Mg^2+^ ions, strong hydrophobic interactions (π-π stackings) with the nucleotide DC_16_ and Pro_145_. Interestingly, compounds **HPCAR-28** and **HPCAR-89** were able to form H-bonds with DG_4_ that can further stabilize the binding mode compared to **HPCAR-142**. Moreover, the fluorine atom of **HPCAR-28** can guarantee additional interactions within the binding site with Pro_145_ and the sidechains of Glu_152_. This slightly different pattern of interactions is also highlighted by the differences in docking scores and ΔG_bind_ values (**HPCAR-28** GlideScore −7.980 kcal/mol and ΔG_bind_ −34.102 kcal/mol; **HPCAR-89** GlideScore −6.648 kcal/mol and ΔG_bind_ −26.777 kcal/mol; **HPCAR-142** −6.622 kcal/mol and ΔG_bind_ −25.759 kcal/mol; as reported in Tables S2, S3). Overall, the *in silico* analysis showed that **HPCAR-28** and **HPCAR-89** can better interact with the active site of HIV IN with respect to **HPCAR-142**.

Regarding the investigation of the binding modes of our derivatives, in our previous study (Sirous et al., 2019), we discussed about the mutation of Tyr_143_ that confers resistance to **RLT**. In particular, **RLT** established interactions with Tyr_143_ by its oxadiazole moiety in both binary PFV-IN and modeled HIV-1 IN complexes. Interestingly, our most promising derivatives do not possess a moiety that can establish interactions with this residue (i.e., oxadiazole in **RLT**). Furthermore, in this study we investigated also two additional mutations that could confer resistance to drugs including **RLT** and **DTG**, Gln_148_His and Gly_140_Ser. The *in silico* analysis reported in Figure S1 showed the superposition of the binding mode of **RLT**, **DTG**, and **HPCAR-28** into HIV-1 IN active site. Notably, the only binding mode that can be strongly influenced by these residues (Tyr_143_, Gln_148_, and Gly_140_) is the one of **RLT**. **DTG** can marginally interact with the mentioned residues, while **HPCAR-28** is largely distant from the residues that are responsible of the resistance (distance from Me of **HPCAR-28** to Tyr_143_ over 5 Å, to Gln_148_ over 9 Å, to Gly_140_ over 10 Å; measured by the measurement tool available in PyMOL). Remarkably, our HPCAR derivatives (**HPCAR-28** and **HPCAR-89**) can additionally target the nucleotide DC16 and DG4 (Figure 4). This analysis is in perfect agreement with the experimental data showing a dramatic decrease of affinity of **RLT** for mutant HIV-1 IN and a lower decrease of affinity of **DTG**. Consequently, it was assumed that the possible mutations of residues Tyr_143_, Gln_148_, and Gly_140_ could not influence the binding of the HPCAR derivatives to IN.

### Molecular Dynamics Simulation Studies

**Figure 5:**
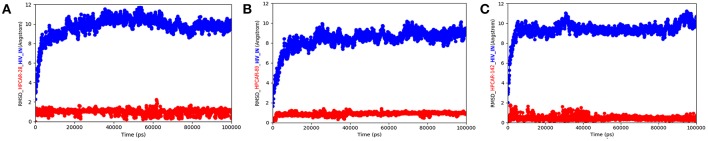
RMSD of the complexes **HPCAR-28**/HIV IN **(A)**, **HPCAR-89**/HIV IN **(B)**, **HPCAR-142**/HIV IN **(C)** (RMSD of the protein in a line formed by blue circles; RMSD of the ligand in a line formed by red circles). RMSD were calculated between the final conformation and the starting conformation through the 100 ns of the MD simulation.

In order to better understand the behavior of the representative compounds into HIV-IN enzyme for providing more reliable results about the interactions of **HPCAR-28**, **HPCAR-89**, and **HPCAR-142** with HIV-IN, we performed MD simulations starting from the docked poses reported in Figure 5 (see Experimental Section for further details).

The three INIs reached an overall stability about after 20 ns. We observed that the pattern of interaction indicated by the docking calculations are generally maintained during the MD, confirming **HPCAR-28** and **HPCAR-89** as more potent potential INIs with respect to the compound **HPCAR-142**. Accordingly, the three compounds maintained the coordination bond with the Mg^2+^ ions during the simulation as well as the hydrophobic interactions with DC_16_ and DA_17_. We also observed that **HPCAR-28** and **HPCAR-89** were able to establish and maintain further contacts into the binding site with respect to the compound **HPCAR-142**. In fact, the presence of a nitrogen in the R_1_ group as in of **HPCAR-28** and **HPCAR-89** allowed to the molecules to stabilize their binding mode by forming further H-bonds with DNA during the simulations. Briefly, the analysis of the computational studies allowed to propose **HPCAR-28** and **HPCAR-89** as more potent INIs with respect to the compound **HPCAR-142**.

In summary, combining different computational techniques for evaluating the affinity of the compounds for HIV IN binding site we provided a more comprehensive *in silico* protocol improving the probability to identify and select compounds with relevant affinity for the selected binding site (Brogi et al., 2009). Accordingly, the presented screening workflow allowed to select novel potential HIV-1 INIs based on HPCAR scaffold with improved predicted pharmacological profile. Taking into account also the synthetic feasibility three representative hit candidates were then chosen for the synthesis and then biologically evaluated for validating the applied computational approach.

### Chemistry

**Figure 6:**
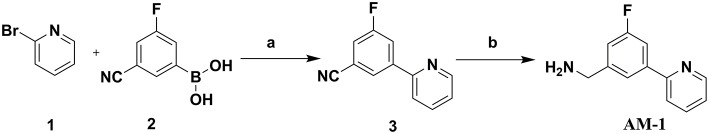
Procedure applied for the synthesis of the amine fragment **AM-1**. Reagents and conditions: (a) Pd(OAc)_2_, PPh_3_, K_2_CO_3_, dioxane/water, 24 h reflux; (b) NaBH_4_, NiCl_2_ (dry), dry ethanol, 25°C.

**Figure 7:**
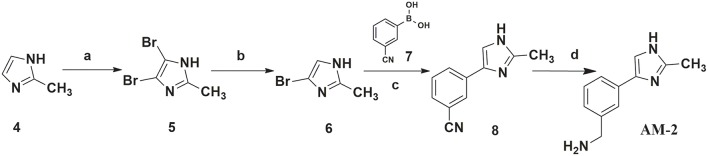
Procedure applied for the synthesis of the amine fragment **AM-2**. Reagents and conditions: (a) Br_2_, chloroform, 0–25°C; (b) Na_2_SO_3_, ethanol/water, 48 h reflux; (c) Pd(OAc)_2_, PPh_3_, K_2_CO_3_, dioxane/water, 24 h reflux (d) NaBH_4_, NiCl_2_ (dry), dry ethanol, 25°C.

**Figure 8:**
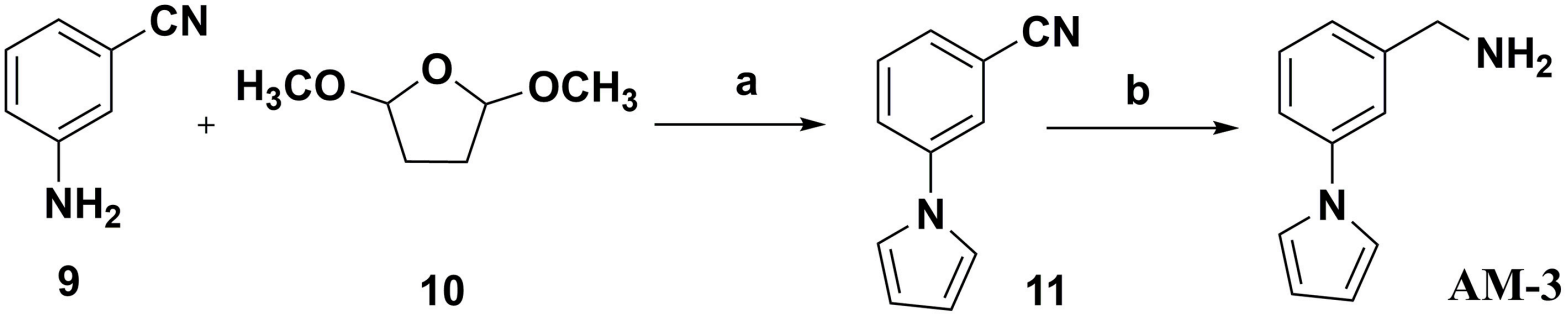
Procedure applied for the synthesis of the amine fragment **AM-3**. Reagents and conditions: (a) 5 N HCl, dioxane, 30 min reflux; (b) NaBH_4_, NiCl_2_ (dry), dry ethanol, 25°C.

**Figure 9:**
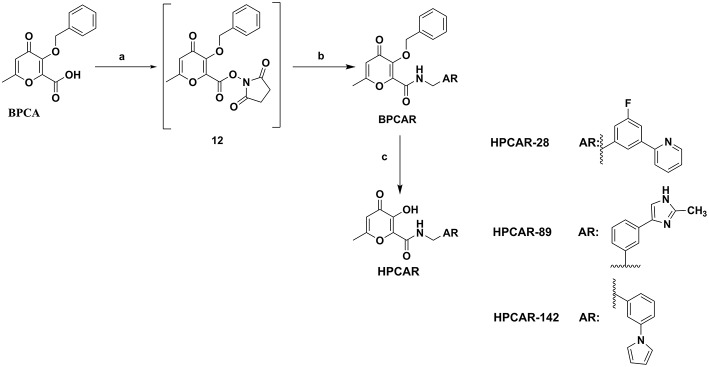
General procedure applied for the final synthesis of three representative hit compounds based on HPCAR scaffold. Reagents and conditions: (a) NHS, EDCI, Dichloromethane (dry), 25°C, 3 h; (b) AR-CH_2_NH_2_ (amine compounds used for **HPCAR-28, HPCAR-89**, and **HPCAR-142** are **AM-1**, **AM-2**, and **AM-3**, respectively), 25°C, 3 days; (c) BBr_3_, Dichloromethane (dry), 0–25°C.

In this study, 3-(benzyloxy)-6-methyl-4-oxo-4*H*-pyran-2-carboxylic acid, **BPCA** was used as a key intermediate material for the preparation of the three selected hit compounds. Thus, this intermediate was first synthesized starting from commercially available Kojic acid according to a synthetic procedure previously employed in our laboratory (Sirous et al., 2019). On the other hand, three different synthetic routes were developed for the preparation of the amine fragments needed for appending desired hydrophobic backbone to **BPCA**. The methodologies adopted for the synthesis of amine compounds **AM-(1-3)** are outlined in Figures 6–9, respectively.

As described in Figure 6, the synthesis of the amine fragment **AM-1** started using an efficient Suzuki–Miyaura cross-coupling (SMC) (Tan et al., 2014) by reacting 2-bromopyridine **1** with phenyl-boronic acid derivative **2** in the presence of tetrakis (tri-phenylphosphine) palladium (0) as the catalyst, affording the phenyl-pyridine derivative **3**. Subsequently the nitrile group of resulting compound **3** was reduced to the corresponding primary amine **AM-1** by treatment with sodium borohydride and dry nickel (II) chloride (Khurana and Gogia, 1997; Khurana and Kukreja, 2002; Caddick et al., 2003).

A four-step synthetic procedure was employed for the synthesis of the amine fragment **AM-2** starting from 2-methyl-imidazole **4** (Figure 7). The first step of the protocol consisted of the double bromination of the imidazole ring which afforded the dibromo-imidazole derivative **5** in a moderate yield. The selective debromination of the vicinal dibromide **5** employing sodium sulfite as the reducing agent in aqueous ethanol at reflux temperature effectively provided product **6** (yield: 75%) (Khurana and Gogia, 1997; Alonso-Alija et al., 2003). Bromo-imidazole **6** underwent a classical SM coupling reaction with phenyl-boronic acid **7**, providing phenyl-imidazole derivative **8**. Amine fragment **AM-2** was finally obtained by reduction of phenyl-imidazole derivative **8** using sodium borohydride and dry nickel (II) chloride as described for **AM-1** (Caddick et al., 2003).

The amine fragment **AM-3** was prepared from commercially available 3-aminobenzonitrile **9**, following Figure 8. A Clauson–Kaas reaction of 3-aminobenzonitrile **9** with dimethoxytetrahydrofuran **10** catalyzed by hydrochloric acid afforded the corresponding 1-phenylpyrrole derivative **11** in a good yield (82%) (Chatzopoulou et al., 2013). The nitrile functionality of the resulting product was then reduced in the presence of sodium borohydride and dry nickel (II) chloride to furnish the desired amine compound **AM-3** (Caddick et al., 2003).

Finally, the representative hit compounds were prepared by introduction of amine backbones to the benzyl-protected pyranone **BPCA** under standard amide coupling conditions (Sheehan et al., 1965; Sirous et al., 2019). The general procedure employed for the synthesis of the final compounds is summarized in Figure 9. The activation of carboxylic acid group of **BPCA** as the corresponding derivative **12** using EDCI and NHS as coupling reagents followed by treatment with the desired amines led to 2-amido substituted pyranone analogs **BPCAR**. The removal of the benzyl protecting group of the obtained amide derivatives was then accomplished by reaction with boron tribromide in dichloromethane at room temperature to obtain three final target products, **HPCAR-28, HPCAR**-**89**, and **HPCAR-142**, in 71–86% yield (Kosak et al., 2015; Sirous et al., 2019).

### Biological Activity Evaluation

For validating the computational protocol herein presented, three representative compounds (**HPCAR-28**, **HPCAR-89**, and **HPCAR-142**) were synthesized and biologically assessed for HIV-1 IN catalytic inhibitory activity based on an *in vitro* enzymatic assay. Given that most HIV-1 INIs such as RLT target the ST step of the integration reaction, the inhibition of the ST activity of HIV-1 IN was examined in these assays in addition to overall HIV-1 IN inhibition. Moreover, assessment of the anti-HIV-1 potential in MT-4 cells was performed in a multiple round cell-based antiviral assay. Cytotoxicity of the selected compounds for the target host cell was also evaluated and their therapeutic indices were calculated. In these experiments, **RLT** was employed as a reference HIV-1 INI. The results for biological activities of these hits were summarized in Table 3.

**Table 3:** Results for anti-HIV activity, inhibitory potential of HIV-1 IN and cytotoxicity of the synthesized compounds and **RLT**.

**Entry**	**Overall HIV-1 IN activity inhibition IC_**50**_ (μM)^a^**	**HIV-1 IN strand transfer activity inhibition IC_**50**_ (μM)^b^**	**HIV-1 activity inhibition EC_**50**_ (μM)^c^**	**Cellular toxicity CC_**50**_ (μM)^d^**	**TI^e^**
**HPCAR-28**	0.065 ± 0.01	3.37 ± 0.38	0.23 ± 0.18	>250	>1,087
**HPCAR-89**	0.27 ± 0.1	4.0 ± 0.34	2.34 ± 0.46	>125	>53.4
**HPCAR-142**	1.97 ± 1.06	17.49 ± 0.13	4.21 ± 0.52	>85.4	>20.3
**HPb**	0.37 ± 0.17	1.405 ± 0.01	1.72 ± 0.99	>250	>145
**HPd**	0.7 ± 0.31	1.54 ± 0.2	1.95 ± 0.93	195.5 ± 4.4	100.3
**RLT**	0.01	0.04	0.013 ± 0.008	>18.329	>1410

a, b *Concentration required to inhibit 50% of the in vitro overall and strand transfer integrase activities, respectively*.

c *Effective concentration in which 50% inhibition is observed*.

d *Cytotoxic concentration in which 50% of the cells are killed*.

e *Therapeutic index: defined by CC_50_/EC_50_ ratio*.

As reported in Table 3, biological evaluation confirmed the favorable anti-HIV profile for the three newly synthesized entities, which demonstrated the ability to inhibit the catalytic activities of HIV-1 IN in the low micromolar range and highlighting the validity of the developed computational protocol for optimizing the previous developed compound. The positive influence of hydrophobic moiety modification of HPCAR derivatives on the inhibitory activity was particularly evident with phenyl-pyridine substituted derivative **HPCAR-28**. This compound emerged as the most potent inhibitor among three tested compounds with low nanomolar activity against HIV-1 IN (IC_50_ = 65 nM) as highlighted by the computational studies, and a 6-fold improvement in anti-IN potency compared to **HPb** (Figure 2 and Table 3; IC_50_ = 0.37 μM) (Sirous et al., 2019). Compounds **HPCAR-89** and **HPCAR-142** also showed promising anti-IN activities in the low micromolar range. In this regard, the incorporation of phenyl-imidazole moiety at the carboxamide sidechain (compound **HPCAR-89**) proved to be also advantageous since it led to a slight enhancement in HIV-1 IN inhibitory activity (IC_50_ = 0.27 μM) compared to the respective *para*-fluorobenzyl amide analog. Although hit compound **HPCAR-142** bearing phenyl-pyrrole fragment was less active than two other hits in HIV-1 IN inhibition (IC_50_ = 1.97 μM), it is still a promising candidate compound for further structural optimization. It was also found that synthesized compounds have the capacity to inhibit the ST step of the HIV-1 IN in the low micromolar range. These observations were in agreement with the *in silico* analysis done, highlighting the fundamental validity of the developed computational protocol for optimizing the previous developed compound. Examination of anti-HIV-1 activity of representative compounds based on cell-based assay indicated that benzyl group replacement with desired hydrophobic moieties was well tolerated for the inhibition of HIV-1 replication as well. According to these results, antiviral activities are reasonably correlated with HIV-1 IN inhibition potencies thus confirming the mechanism of action of these anti-HIV-1 agents. Accordingly, **HPCAR-28** showed the best anti-IN activity and it is the most active hit against HIV-1 infected cells with an EC_50_ value of 0.23 μM.

The cytotoxicity assay also showed that the tested compounds are safe and possess anti-HIV activity at non-cytotoxic concentrations (CC_50_ values ranging from 85.4 to >250 μM), thus resulting in favorable therapeutic indices for the investigated compounds. In particular, the most promising hit compound (**HPCAR-28**) revealed an appreciable therapeutic index (TI > 1,087) comparable to that found for RLT (TI > 1,410). On the contrary, the limited toxicity (>85.4 μM) showed by **HPCAR-142** is potentially ascribable to the presence of the pyrrole moiety that often presents some toxicity.

Overall, these results confirmed that three representative hit compounds are able to achieve the desired level of biological activities in terms of reduced toxicity and optimum inhibitory activities against HIV-1 IN and HIV-1 in cell culture. Furthermore, it was clearly verified that modification of the hydrophobic aromatic moiety within the HPCAR derivatives can lead to differences in HIV-1 IN inhibitory profiles. Moreover, this research clearly confirms the key role of the *in silico* drug design in medicinal chemistry to optimize compounds for a selected binding site. Remarkably the presented protocol could be easily translated to different targets in order to find suitable decoration for optimizing promising hit compounds.

## Conclusion

In the present study, we have reported the development of a computational protocol for identifying novel analogs based on recently disclosed 3-hydroxyl-pyran-4-one-2-carboxamides (HPCAR) scaffold (Sirous et al., 2019) with improved activity against HIV-1 IN. In particular, the *in silico* protocol allowed us to replace the aromatic hydrophobic moiety of HPCAR with appropriate hydrophobic aromatic/hetero-aromatic fragments. To this end, we used a combinatorial side chain hopping strategy. The resulting compounds (>37,000) were filtered using different computational methodologies. Filtering criteria included: appropriate calculated physico-chemical properties, satisfactory docking score values, visual inspection, lower ligand binding energies and proper behaviors into the HIV IN binding site assessed by MD. By using these subsequent filtering tools, we reduced the number of compounds from 1,803 to 40. Among the 40 top hit compounds, three HPCAR derivatives were chosen according to the relevant computational outputs coupled to a synthetic accessibility. After the synthesis of **HPCAR-28**, **HPCAR-89**, and **HPCAR-142**, the compounds underwent to biological evaluation in order to validate the described *in silico* protocol. Gratifyingly, the results of pharmacological studies showed that the representative hit compounds inhibited HIV-1 IN in the low micromolar range. Among them, compound **HPCAR-28** showed the best inhibitory activity against HIV-1 IN as well as the best inhibitory activity against HIV-1 replication and HIV-1 IN strand transfer process along with a notable therapeutic index and no appreciable cell toxicity.

These promising and encouraging results provide further solid support for the potential exploitation of HPCAR scaffold in the development of anti-retroviral drugs, paving the way to the discovery of a new class of drugs against HIV-1 IN for treating HIV infection.

## Data Availability

All datasets generated for this study are included in the manuscript/Supplementary Files.

## Author Contributions

HS carried out the computational experiments and the synthesis of the selected compounds, contributing in writing the manuscript. GCh carried out the computational experiments and performed the acquisition, analysis, interpretation of data, contributing in writing, and revising the manuscript. SG and SBu advised in the synthesis of the compounds, contributing in revising the manuscript. ZD and FC performed the biological evaluation of the selected compounds. LS advised in the synthesis of the compounds. SBr conceived, designed, and performed the computational experiments, supervised the overall work, wrote and revised the manuscript. AF advised in the synthesis of the compounds and in the computational experiments, contributing in revising the manuscript. GCa supervised the overall work and contributing in revising the manuscript. MB conceived the synthetic strategy for the synthesis of the compounds and supervised the synthesis of them, contributing in revising the manuscript.

### Conflict of Interest Statement

The authors declare that the research was conducted in the absence of any commercial or financial relationships that could be construed as a potential conflict of interest. The handling editor declared a shared affiliation, though no other collaboration, with one of the authors MB.
