# Supplementary material for: Identification of Novel 3-Hydroxy-pyran-4-One Derivatives as Potent HIV-1 Integrase Inhibitors Using in silico Structure-Based Combinatorial Library Design Approach
Source: Front Chem. 2019 Aug 13;7:574. doi: 10.3389/fchem.2019.00574 (PMC6700280; doi:10.3389/fchem.2019.00574)
Supplement: Supplementary file 1 [file Table_1.DOCX]

Supplementary Material

Identification of novel 3-hydroxy-pyran-4-one derivatives as potent HIV-1 integrase inhibitors using *in silico* structure-based combinatorial library design approach

Hajar Sirous^1+^, Giulia Chemi^2+^, Sandra Gemma^2^, Stefania Butini^2^, Zeger Debyser^3^, Frauke Christ^3^, Lotfollah Saghaie^4^, Simone Brogi^5*^, Afshin Fassihi^4**^, Giuseppe Campiani^2***^, Margherita Brindisi^6^

^1^Bioinformatics Research Center, School of Pharmacy and Pharmaceutical Sciences, Isfahan University of Medical Sciences, Isfahan, Iran

^2^Department of Biotechnology, Chemistry and Pharmacy, Department of Excellence 2018-2022, University of Siena, via Aldo Moro 2, 53100 Siena, Italy

^3^Molecular Medicine, K.U. Leuven and IRC KULAK, Kapucijnenvoer 33, B-3000 Leuven, Flanders, Belgium

^4^Department of Medicinal Chemistry, Faculty of Pharmacy, Isfahan University of Medical Sciences, 81746-73461 Isfahan, Iran

^5^Department of Pharmacy, University of Pisa, via Bonanno 6, 56126, Pisa, Italy

^6^Department of Pharmacy, Department of Excellence 2018-2022, University of Naples Federico II, via D. Montesano 49, 80131 Naples, Italy

^+^These authors equally contributed to this work

**Correspondence:**Corresponding Authors
*[simone.brogi@unipi.it](mailto:simone.brogi@unipi.it)

**[fassihi@pharm.mui.ac.ir](mailto:fassihi@pharm.mui.ac.ir)

[***campiani@unisi.it](mailto:***campiani@unisi.it)

**Table of Contents**

**Table S1 page S2**

**Table S2 page S25**

**Table S3 page S36**

**Figure S1 page S39**

**NMR spectra page S40**

**Table S1.** The ADMET prediction results of 144 top screened combinatorial hits obtained through aromatic group variation. The selected physicochemical properties were calculated using QikProp module of Schrödinger.

| **entry** | **Structure** | **QPlogP^a^** | **QPlogHERG^b^** | **QPPCaco^c^** | **QPlogBB^d^** | **QPPMDCK^e^** | **Rule of five^f^** |
| --- | --- | --- | --- | --- | --- | --- | --- |
| **HPCAR-1** |  | 3.180 | -6.843 | 227.188 | -1.552 | 99.696 | 0 |
| **HPCAR-2** |  | 2.545 | -6.808 | 104.382 | -1.015 | 47.586 | 0 |
| **HPCAR-3** |  | 3.062 | -6.784 | 298.176 | -1.628 | 133.757 | 0 |
| **HPCAR-4** |  | 2.559 | -6.783 | 134.086 | -1.826 | 56.384 | 0 |
| **HPCAR-5** |  | 2.944 | -6.783 | 270.203 | -1.681 | 120.247 | 0 |
| **HPCAR-6** |  | 4.108 | -6.642 | 478.440 | -1.200 | 313.636 | 0 |
| **HPCAR-7** |  | 2.647 | -6.633 | 211.354 | -1.761 | 92.207 | 0 |
| **HPCAR-8** |  | 2.283 | -6.569 | 157.367 | -1.680 | 67.036 | 0 |
| **HPCAR-9** |  | 3.335 | -6.533 | 408.068 | -1.195 | 187.758 | 0 |
| **HPCAR-10** |  | 3.177 | -6.529 | 310.514 | -1.567 | 139.750 | 0 |
| **HPCAR-11** |  | 3.965 | -6.529 | 422.271 | -1.232 | 194.831 | 0 |
| **HPCAR-12** |  | 3.372 | -6.513 | 257.420 | -1.510 | 114.110 | 0 |
| **HPCAR-13** |  | 2.349 | -6.503 | 110.208 | -1.916 | 45.614 | 0 |
| **HPCAR-14** |  | 3.845 | -6.496 | 344.411 | -1.482 | 156.309 | 0 |
| **HPCAR-15** |  | 3.590 | -6.481 | 489.859 | -1.263 | 269.009 | 0 |
| **HPCAR-16** |  | 3.355 | -6.449 | 261.880 | -1.454 | 116.249 | 0 |
| **HPCAR-17** |  | 3.649 | -6.421 | 354.720 | -1.357 | 278.567 | 0 |
| **HPCAR-18** |  | 2.345 | -6.418 | 202.856 | -1.732 | 88.207 | 0 |
| **HPCAR-19** |  | 3.591 | -6.404 | 412.720 | -1.206 | 190.073 | 0 |
| **HPCAR-20** |  | 1.958 | -6.337 | 112.965 | -0.894 | 51.829 | 0 |
| **HPCAR-21** |  | 2.000 | -6.328 | 109.338 | -0.750 | 50.033 | 0 |
| **HPCAR-22** |  | 3.280 | -6.321 | 255.705 | -1.447 | 113.288 | 0 |
| **HPCAR-23** |  | 2.517 | -6.315 | 194.236 | -1.663 | 84.162 | 0 |
| **HPCAR-24** |  | 2.381 | -6.284 | 125.048 | -1.891 | 52.287 | 0 |
| **HPCAR-25** |  | 2.272 | -6.262 | 98.838 | -0.709 | 81.183 | 0 |
| **HPCAR-26** |  | 2.572 | -6.259 | 106.149 | -1.911 | 75.498 | 0 |
| **HPCAR-27** |  | 2.064 | -6.239 | 218.761 | -1.576 | 95.705 | 0 |
| **HPCAR-28** |  | 3.309 | -6.346 | 364.631 | -1.214 | 298.516 | 0 |
| **HPCAR-29** |  | 1.863 | -6.214 | 112.044 | -0.851 | 51.373 | 0 |
| **HPCAR-30** |  | 2.213 | -6.210 | 120.881 | -0.725 | 55.731 | 0 |
| **HPCAR-31** |  | 3.209 | -6.186 | 407.138 | -1.114 | 338.354 | 0 |
| **HPCAR-32** |  | 2.970 | -6.174 | 298.230 | -1.497 | 183.582 | 0 |
| **HPCAR-33** |  | 2.929 | -6.173 | 339.732 | -1.181 | 258.856 | 0 |
| **HPCAR-34** |  | 2.474 | -6.157 | 231.132 | -1.564 | 101.568 | 0 |
| **HPCAR-35** |  | 2.225 | -6.155 | 260.820 | -1.316 | 200.249 | 0 |
| **HPCAR-36** |  | 2.393 | -6.141 | 97.050 | -0.642 | 107.665 | 0 |
| **HPCAR-37** |  | 3.831 | -6.134 | 499.493 | -1.002 | 421.560 | 0 |
| **HPCAR-38** |  | 2.067 | -6.120 | 205.810 | -1.499 | 89.596 | 0 |
| **HPCAR-39** |  | 2.848 | -6.109 | 249.926 | -1.472 | 110.524 | 0 |
| **HPCAR-40** |  | 3.334 | -6.097 | 321.998 | -1.373 | 145.344 | 0 |
| **HPCAR-41** |  | 2.013 | -6.093 | 126.159 | -0.751 | 58.402 | 0 |
| **HPCAR-42** |  | 1.843 | -6.090 | 142.439 | -1.731 | 60.190 | 0 |
| **HPCAR-43** |  | 2.421 | -6.075 | 103.073 | -0.719 | 46.941 | 0 |
| **HPCAR-44** |  | 3.518 | -6.074 | 355.945 | -1.229 | 161.975 | 0 |
| **HPCAR-45** |  | 2.120 | -6.073 | 206.418 | -1.487 | 89.882 | 0 |
| **HPCAR-46** |  | 2.679 | -6.065 | 149.130 | -1.715 | 105.012 | 0 |
| **HPCAR-47** |  | 3.188 | -6.058 | 244.829 | -1.403 | 211.024 | 0 |
| **HPCAR-48** |  | 2.653 | -6.035 | 232.196 | -1.444 | 102.074 | 0 |
| **HPCAR-49** |  | 2.491 | -6.029 | 258.606 | -1.510 | 114.678 | 0 |
| **HPCAR-50** |  | 2.519 | -6.010 | 353.194 | -1.202 | 249.289 | 0 |
| **HPCAR-51** |  | 1.884 | -6.009 | 198.031 | -1.431 | 148.146 | 0 |
| **HPCAR-52** |  | 3.804 | -7.000 | 323.728 | -1.457 | 146.188 | 0 |
| **HPCAR-53** |  | 2.863 | -6.932 | 247.618 | -1.811 | 109.421 | 0 |
| **HPCAR-54** |  | 3.765 | -5.726 | -6.828 | 385.738 | -1.346 | 0 |
| **HPCAR-55** |  | 3.047 | -6.765 | 233.967 | -1.882 | 102.915 | 0 |
| **HPCAR-56** |  | 2.082 | -6.660 | 110.379 | -2.002 | 45.690 | 0 |
| **HPCAR-57** |  | 3.163 | -6.653 | 240.476 | -1.815 | 106.014 | 0 |
| **HPCAR-58** |  | 2.695 | -6.591 | 104.931 | -2.028 | 43.257 | 0 |
| **HPCAR-59** |  | 2.194 | -6.566 | 102.213 | -0.926 | 46.518 | 0 |
| **HPCAR-60** |  | 3.141 | -6.549 | 128.419 | -2.086 | 53.812 | 0 |
| **HPCAR-61** |  | 2.748 | -6.538 | 122.652 | -2.128 | 51.206 | 0 |
| **HPCAR-62** |  | 2.969 | -6.501 | 130.925 | -1.930 | 87.528 | 0 |
| **HPCAR-63** |  | 2.683 | -6.400 | 169.413 | -1.820 | 72.599 | 0 |
| **HPCAR-64** |  | 2.667 | -6.399 | 163.135 | -1.844 | 69.696 | 0 |
| **HPCAR-65** |  | 2.855 | -6.392 | 186.593 | -1.774 | 80.589 | 0 |
| **HPCAR-66** |  | 2.845 | -6.388 | 194.163 | -1.752 | 84.129 | 0 |
| **HPCAR-67** |  | 2.712 | -6.386 | 120.341 | -2.012 | 50.163 | 0 |
| **HPCAR-68** |  | 3.177 | -6.358 | 367.805 | -1.379 | 167.817 | 0 |
| **HPCAR-69** |  | 2.892 | -6.323 | 308.615 | -1.523 | 138.826 | 0 |
| **HPCAR-70** |  | 3.398 | -6.298 | 297.925 | -1.629 | 191.227 | 0 |
| **HPCAR-71** |  | 2.549 | -6.281 | 121.767 | -0.800 | 56.208 | 0 |
| **HPCAR-72** |  | 2.478 | -6.279 | 118.219 | -1.912 | 66.211 | 0 |
| **HPCAR-73** |  | 3.042 | -6.273 | 211.970 | -1.630 | 141.093 | 0 |
| **HPCAR-74** |  | 2.169 | -6.273 | 152.213 | -1.761 | 64.666 | 0 |
| **HPCAR-75** |  | 3.082 | -6.244 | 199.862 | -1.636 | 156.630 | 0 |
| **HPCAR-76** |  | 2.684 | -6.239 | 97.481 | -0.819 | 107.860 | 0 |
| **HPCAR-77** |  | 2.547 | -6.212 | 222.917 | -1.553 | 97.672 | 0 |
| **HPCAR-78** |  | 2.510 | -6.207 | 104.726 | -0.868 | 47.756 | 0 |
| **HPCAR-79** |  | 2.742 | -6.190 | 232.926 | -1.490 | 190.271 | 0 |
| **HPCAR-80** |  | 3.697 | -6.176 | 430.863 | -1.223 | 199.120 | 0 |
| **HPCAR-81** |  | 4.258 | -6.158 | 454.138 | -1.161 | 210.771 | 0 |
| **HPCAR-82** |  | 3.040 | -6.140 | 324.331 | -1.418 | 146.483 | 0 |
| **HPCAR-83** |  | 3.189 | -6.118 | 445.398 | -1.134 | 302.663 | 0 |
| **HPCAR-84** |  | 2.540 | -6.085 | 102.513 | -1.919 | 74.668 | 0 |
| **HPCAR-85** |  | 3.047 | -6.055 | 282.205 | -1.470 | 174.137 | 0 |
| **HPCAR-86** |  | 2.465 | -6.046 | 171.769 | -1.690 | 73.691 | 0 |
| **HPCAR-87** |  | 2.198 | -6.031 | 264.287 | -1.437 | 117.404 | 0 |
| **HPCAR-88** |  | 2.941 | -6.003 | 326.682 | -1.248 | 372.999 | 0 |
| **HPCAR-89** |  | 2.465 | -5.988 | 200.574 | -1.565 | 87.135 | 0 |
| **HPCAR-90** |  | 3.366 | -5.734 | 455.270 | -1.203 | 211.339 | 0 |
| **HPCAR-91** |  | 2.730 | -5.805 | 425.420 | -1.144 | 196.402 | 0 |
| **HPCAR-92** |  | 2.665 | -5.275 | 368.283 | -0.975 | 290.417 | 0 |
| **HPCAR-93** |  | 3.495 | -5.122 | 565.382 | -0.635 | 2729.104 | 0 |
| **HPCAR-94** |  | 2.384 | -5.772 | 222.123 | -1.404 | 97.296 | 0 |
| **HPCAR-95** |  | 3.017 | -5.215 | 474.906 | -0.887 | 787.977 | 0 |
| **HPCAR-96** |  | 2.662 | -5.573 | 347.697 | -1.220 | 157.922 | 0 |
| **HPCAR-97** |  | 2.016 | -5.325 | 162.447 | -1.373 | 149.305 | 0 |
| **HPCAR-98** |  | 2.344 | -5.785 | 303.124 | -1.246 | 136.158 | 0 |
| **HPCAR-99** |  | 2.939 | -5.995 | 349.909 | -1.107 | 321.209 | 0 |
| **HPCAR-100** |  | 2.675 | -5.780 | 335.850 | -1.247 | 145.114 | 0 |
| **HPCAR-101** |  | 3.345 | -5.272 | 381.310 | -0.848 | 663.048 | 0 |
| **HPCAR-102** |  | 2.648 | -5.747 | 364.467 | -1.209 | 166.171 | 0 |
| **HPCAR-103** |  | 2.352 | -5.789 | 235.569 | -1.437 | 103.677 | 0 |
| **HPCAR-104** |  | 2.791 | -5.807 | 341.236 | -1.078 | 342.795 | 0 |
| **HPCAR-105** |  | 2.628 | -5.645 | 332.180 | -1.208 | 150.318 | 0 |
| **HPCAR-106** |  | 2.210 | -5.573 | 255.545 | -1.416 | 113.212 | 0 |
| **HPCAR-107** |  | 2.602 | -5.726 | 291.826 | -1.303 | 130.681 | 0 |
| **HPCAR-108** |  | 2.737 | -5.756 | 411.971 | -1.121 | 189.700 | 0 |
| **HPCAR-109** |  | 3.245 | -5.632 | 421.846 | -1.190 | 194.619 | 0 |
| **HPCAR-110** |  | 3.250 | -5.480 | 428.373 | -1.120 | 197.876 | 0 |
| **HPCAR-111** |  | 2.957 | -5.577 | 456.222 | -1.287 | 211.817 | 0 |
| **HPCAR-112** |  | 2.566 | -5.715 | 324.686 | -1.126 | 249.456 | 0 |
| **HPCAR-113** |  | 3.622 | -5.326 | 566.239 | -0.961 | 267.530 | 0 |
| **HPCAR-114** |  | 2.752 | -5.529 | 273.946 | -1.420 | 122.048 | 0 |
| **HPCAR-115** |  | 2.479 | -5.985 | 280.009 | -1.468 | 124.971 |  |
| **HPCAR-116** |  | 2.377 | -5.949 | 227.139 | -1.486 | 99.673 | 0 |
| **HPCAR-117** |  | 2.154 | -5.606 | 333.308 | -1.248 | 150.870 | 0 |
| **HPCAR-118** |  | 2.934 | -5.568 | 412.937 | -1.201 | 190.180 | 0 |
| **HPCAR-119** |  | 3.363 | -5.751 | 554.177 | -1.121 | 261.375 | 0 |
| **HPCAR-120** |  | 2.627 | -5.372 | 495.839 | -0.972 | 540.108 | 0 |
| **HPCAR-121** |  | 2.316 | -5.719 | 307.878 | -1.299 | 138.467 | 0 |
| **HPCAR-122** |  | 3.255 | -5.557 | 252.910 | -1.533 | 111.951 | 0 |
| **HPCAR-123** |  | 2.132 | -5.835 | 167.329 | -1.673 | 71.634 | 0 |
| **HPCAR-124** |  | 2.120 | -5.716 | 255.225 | -1.411 | 113.059 | 0 |
| **HPCAR-125** |  | 2.400 | -5.516 | 266.322 | -1.237 | 243.132 | 0 |
| **HPCAR-126** |  | 2.532 | -5.614 | 210.327 | -1.358 | 219.439 | 0 |
| **HPCAR-127** |  | 3.059 | -5.826 | 260.998 | -1.305 | 241.584 | 0 |
| **HPCAR-128** |  | 2.641 | -5.447 | 439.943 | -1.091 | 203.659 | 0 |
| **HPCAR-129** |  | 2.341 | -5.577 | 246.536 | -1.436 | 108.904 | 0 |
| **HPCAR-130** |  | 2.808 | -5.406 | 324.683 | -1.306 | 146.655 | 0 |
| **HPCAR-131** |  | 3.518 | -5.760 | 422.239 | -1.536 | 194.815 | 0 |
| **HPCAR-132** |  | 3.158 | -5.515 | 406.917 | -1.358 | 187.185 | 0 |
| **HPCAR-133** |  | 2.256 | -5.998 | 237.251 | -1.402 | 187.394 | 0 |
| **HPCAR-134** |  | 3.646 | -5.325 | 374.573 | -1.352 | 171.156 | 0 |
| **HPCAR-135** |  | 2.683 | -5.916 | 281.308 | -1.368 | 125.598 | 0 |
| **HPCAR-136** |  | 2.958 | -5.852 | 394.252 | -1.222 | 180.896 | 0 |
| **HPCAR-137** |  | 2.524 | -5.604 | 306.124 | -1.378 | 137.615 | 0 |
| **HPCAR-138** |  | 3.485 | -5..932 | 435.829 | -1.087 | 328.738 | 0 |
| **HPCAR-139** |  | 2.805 | -5.778 | 384.135 | -1.292 | 175.884 | 0 |
| **HPCAR-140** |  | 3.721 | -5.432 | 639.126 | -1.046 | 304.939 | 0 |
| **HPCAR-141** |  | 3.033 | -5.708 | 229.669 | -1.353 | 200.832 | 0 |
| **HPCAR-142** |  | 3.129 | -6.240 | 457.091 | -1.108 | 212.253 | 0 |
| **HPCAR-143** |  | 3.024 | -5.815 | 418.792 | -1.209 | 193.097 | 0 |
| **HPCAR-144** |  | 1.850 | -6.126 | 130.651 | -0.828 | 60.653 | 0 |

^a^QPlogP predicted octanol/water partition coefficient (acceptable range or recommended value for 95% of known drugs -2.0-6.5); ^b^QPlogHERG predicted IC_50_ value for blockage of HERG K^+^ channels (acceptable range or recommended below -5.0); ^c^QPPCaco predicted apparent Caco-2 cell permeability in nm/sec. Caco-2 cells are a model for the gut-blood barrier. QikProp predictions are for non-active transport (acceptable range or recommended value for 95% of known drugs: <25 is poor and >500 is great);^d^QPlogBB predicted brain/blood partition coefficient (acceptable range or recommended value for 95% of known drugs -3-1.2); ^e^QPPMDCK predicted apparent MDCK cell permeability in nm/sec. MDCK cells are considered to be a good mimic for the blood-brain barrier. QikProp predictions are for non-active transport (acceptable range or recommended value for 95% of known drugs :< 25 is poor and >500 is great); ^f^predicted number of violations of Lipinski’s rule of five. The rules are: MW < 500, QPlogP < 5, donorHB ≤ 5, accptHB ≤ 10 (acceptable range or recommended value is maximum 4).

**Table S2.** The Quantum polarized ligand docking results of 76 top screened combinatorial with active site of HIV-1 IN model

| **entry** | **XP Glide Score** | **Docking emodel** | **Interaction with Mg^2+^** | **Interacting amino acide residues** | **Interacting nucleotide residues** |
| --- | --- | --- | --- | --- | --- |
| **HPCAR-1** | -6.103 | -81.633 | 4-C=O...Mg^2+^(2.18)***^b^***  3-OH…Mg^2+^  (2.10 and 2.49)  NHC=O…Mg^2+^(2.05) | Hydrophobic: Tyr_143_,Pro_145,_ Gln_146_ | H-bonding:DG_4_  π-π: DC_16_ |
| **HPCAR-2** | -7.572 | -66.671 | 4-C=O...Mg^2+^(2.12)  3-OH…Mg^2+^  (2.97 and 2.08)  NHC=O…Mg^2+^(2.06) | Hydrophobic: Tyr_143_,Pro_145,_ Gln_146_ | H-bonding:DG_4_, DC_16_ |
| **HPCAR-3** | -7.043 | -79.537 | 4-C=O...Mg^2+^(2.14)  3-OH…Mg^2+^  (2.09 and 2.16)  NHC=O…Mg^2+^(2.12) | Hydrophobic: Tyr_143_,Pro_145,_ Gln_146_ | H-bonding:DC_16_  π-π: DC_16_ |
| **HPCAR-6** | -6.180 | -69.333 | 4-C=O...Mg^2+^(2.10)  3-OH…Mg^2+^  (2.96 and 2.17)  NHC=O…Mg^2+^(2.12) | Hydrophobic: Tyr_143_,Pro_145,_ Gln_146_ | π-π: DC_16_ |
| **HPCAR-7** | -6.377 | -84.971 | 4-C=O...Mg^2+^(2.18)  3-OH…Mg^2+^  (2.85 and 2.10)  NHC=O…Mg^2+^(2.05) | Hydrophobic: Tyr_143_,Pro_145,_ Gln_146_ | H-bonding:DC_16_  π-π: DC_16_ |
| **HPCAR-8** | -6.155 | -79.277 | 4-C=O...Mg^2+^(2.20)  3-OH…Mg^2+^  (2.45 and 2.24)  NHC=O…Mg^2+^(2.06) | Hydrophobic: Tyr_143_,Pro_145,_ Gln_146_ | H-bonding:DA_17_  π-π: DC_16_ |
| **HPCAR-9** | -6.602 | -78.955 | 4-C=O...Mg^2+^(2.15)  3-OH…Mg^2+^  (2.52 and 2.16)  NHC=O…Mg^2+^(2.09) | Hydrophobic: Tyr_143_,Pro_145,_ Gln_146_ | H-bonding:DA_17_  π-π: DC_16_ |
| **HPCAR-10** | -6.684 | -75.195 | 4-C=O...Mg^2+^(2.10)  3-OH…Mg^2+^  (2.82 and 2.16)  NHC=O…Mg^2+^(2.07) | Hydrophobic: Tyr_143_,Pro_145,_ Gln_146_ | H-bonding: DC16, DA17  π-π: DC_16_ |
| **HPCAR-14** | -6.522 | -75.802 | 4-C=O...Mg^2+^(2.09)  3-OH…Mg^2+^  (2.90 and 2.27)  NHC=O…Mg^2+^(2.02) | Hydrophobic: Tyr_143_,Pro_145,_ Gln_146_ | H-bonding: DA17  π-π: DC_16_ |
| **HPCAR-15** | -6.893 | -76.935 | 4-C=O...Mg^2+^(2.13)  3-OH…Mg^2+^  (2.94 and 2.22)  NHC=O…Mg^2+^(2.16) | Hydrophobic: Tyr_143_,Pro_145,_ Gln_146_ | H-bonding: DC16, DA17  π-π: DC_16_ |
| **HPCAR-17** | -6.141 | -73.937 | 4-C=O...Mg^2+^(2.12)  3-OH…Mg^2+^  (2.93 and 2.21)  NHC=O…Mg^2+^(2.08) | Hydrophobic: Tyr_143_, Pro_145,_ Gln_146_ | H-bonding: DA17  π-π: DC_16_ |
| **HPCAR-20** | -6.061 | -50.398 | 4-C=O...Mg^2+^(2.11)  3-OH…Mg^2+^  (2.85 and 2.15)  NHC=O…Mg^2+^(2.03) | Hydrophobic: Tyr_143_,Pro_145_, Gln_146_, Ser_153_ | π-π: DC_16_ |
| **HPCAR-21** | -6.118 | -75.864 | 4-C=O...Mg^2+^(2.10)  3-OH…Mg^2+^  (2.87 and 2.25)  NHC=O…Mg^2+^(2.11) | Hydrophobic: Tyr_143_,Pro_145_, Gln_146_, Gly_149_ | H-bonding: DA17  π-π: DC_16_ |
| **HPCAR-22** | -6.137 | -76.861 | 4-C=O...Mg^2+^(1.90)  3-OH…Mg^2+^  (2.95 and 2.28)  NHC=O…Mg^2+^(2.00) | Hydrophobic: Tyr_143_,Pro_145_, Gln_146_, Gly_149_ | π-π: DC_16_ |
| **HPCAR-23** | -7.425 | -79.694 | 4-C=O...Mg^2+^(1.98)  3-OH…Mg^2+^  (2.85 and 2.40)  NHC=O…Mg^2+^(2.03) | H-bonding: Gln_146_  Hydrophobic: Tyr_143_,Pro_145_, Gln_146_, Gly_149_ | H-bonding: DG_4_  π-π: DG_4_, DC_16_ |
| **HPCAR-25** | -6.107 | -75.644 | 4-C=O...Mg^2+^(2.11)  3-OH…Mg^2+^  (2.87 and 2.25)  NHC=O…Mg^2+^(2.11) | Hydrophobic: Tyr_143_,Pro_145_, Gln_146_, Gly_149_ | H-bonding: DA17  π-π: DC_16_ |
| **HPCAR-26** | -6.999 | -80.833 | 4-C=O...Mg^2+^(2.16)  3-OH…Mg^2+^  (2.83 and 2.13)  NHC=O…Mg^2+^(2.13) | Hydrophobic: Tyr_143_,Pro_145_, Gln_146_, Gly_149_ | H-bonding: DA_17_  π-π: DC_16_ |
| **HPCAR-28** | -7.980 | -72.349 | 4-C=O...Mg^2+^(2.17)  3-OH…Mg^2+^  (2.58 and 2.19)  NHC=O…Mg^2+^(2.20) | Hydrophobic: Tyr_143_,Pro_145_, Gln_146_, Gly_149_ | H-bonding: DG_4_  π-π: DC_16_ |
| **HPCAR-29** | -6.570 | -70.381 | 4-C=O...Mg^2+^(2.16)  3-OH…Mg^2+^  (2.55 and 2.29)  NHC=O…Mg^2+^(2.04) | Hydrophobic: Tyr_143_,Pro_145_, Gln_146_, Gly_149_ | H-bonding: DA_17_  π-π: DC_16_ |
| **HPCAR-30** | -6.745 | -69.304 | 4-C=O...Mg^2+^(2.67)  3-OH…Mg^2+^  (2.26 and 2.18)  NHC=O…Mg^2+^(1.98) | H-bonding: Pro_145_  Hydrophobic: Tyr_143_,Pro_145_, Gln_146_, Gly_149_ | H-bonding: DA_17_  π-π: DG_4_, DC_16_, DA_17_ |
| **HPCAR-32** | -6.196 | -76.603 | 4-C=O...Mg^2+^(2.87)  3-OH…Mg^2+^  (2.51 and 2.11)  NHC=O…Mg^2+^(2.08) | Hydrophobic: Tyr_143_,Pro_145_, Gln_146_, Gly_149_ | H-bonding: DC_16_  π-π: DC_16_ |
| **HPCAR-33** | -7.380 | -70.951 | 4-C=O...Mg^2+^(2.95)  3-OH…Mg^2+^  (2.48 and 2.15)  NHC=O…Mg^2+^(2.10) | Hydrophobic: Tyr_143_,Pro_145_, Gln_146_ | H-bonding: DG_4_  π-π: DC_16_ |
| **HPCAR-35** | -7.133 | -69.407 | 4-C=O...Mg^2+^(2.16)  3-OH…Mg^2+^  (2.98 and 2.46)  NHC=O…Mg^2+^(1.99) | Hydrophobic: Tyr_143_,Pro_145_, Gln_146_ | H-bonding: DG_4_  π-π: DC_16_ |
| **HPCAR-37** | -6.110 | -79.472 | 4-C=O...Mg^2+^(1.92)  3-OH…Mg^2+^  (2.43 and 2.14)  NHC=O…Mg^2+^(2.07) | Hydrophobic: Tyr_143_,Pro_145_, Gln_146_, Gly_149_ | π-π: DC_16_ |
| **HPCAR-40** | -7.671 | -78.971 | 4-C=O...Mg^2+^(2.08)  3-OH…Mg^2+^  (2.21 and 2.70)  NHC=O…Mg^2+^(2.67) | H-bonding: Asn_117_, Glu_152_  Hydrophobic: Tyr_143_,Pro_145_, Pro_142_, Ser_119_, Gly_118_ | π-π: DA_17_ |
| **HPCAR-41** | -6.658 | -66.409 | 4-C=O...Mg^2+^(2.12)  3-OH…Mg^2+^  (2.97 and 2.12)  NHC=O…Mg^2+^(2.05) | Hydrophobic: Tyr_143_,Pro_145_, Gln_146_, Gly_149_ | π-π: DC_16_ |
| **HPCAR-42** | -6.651 | -69.593 | 4-C=O...Mg^2+^(1.99)  3-OH…Mg^2+^  (2.85 and 2.49)  NHC=O…Mg^2+^(2.01) | H-bonding: Gln_146_  Hydrophobic: Tyr_143_,Pro_145_, Gln_146_ | π-π: DC_16_ |
| **HPCAR-44** | -6.640 | -80.299 | 4-C=O...Mg^2+^(2.62)  3-OH…Mg^2+^  (2.51 and 2.24)  NHC=O…Mg^2+^(2.06) | Hydrophobic: Tyr_143_,Pro_145_, Gln_146_, Gly_149_ | H-bonding: DA_17_  π-π: DC_16_ |
| **HPCAR-45** | -6.170 | -67.516 | 4-C=O...Mg^2+^(2.74)  3-OH…Mg^2+^  (2.49 and 2.14)  NHC=O…Mg^2+^(2.05) | Hydrophobic: Tyr_143_,Pro_145_, Gln_146_, Gly_149_ | H-bonding: DG_4,_ DA_17_  π-π: DC_16_ |
| **HPCAR-46** | -7.302 | -76.331 | 4-C=O...Mg^2+^(2.77)  3-OH…Mg^2+^  (2.42 and 2.18)  NHC=O…Mg^2+^(2.04) | H-bonding: Gln_146_  Hydrophobic: Tyr_143_,Pro_145_, Gln_146_, Gly_149_ | H-bonding: DG_4_  π-π: DC_16_ |
| **HPCAR-52** | -6.392 | -92.314 | 4-C=O...Mg^2+^(2.77)  3-OH…Mg^2+^  (2.42 and 2.18)  NHC=O…Mg^2+^(2.04) | H-bonding: Gln_146_  Hydrophobic: Tyr_143_,Pro_145_, Gln_146_, Gly_149_ | H-bonding: DG_4_  π-π: DC_16_ |
| **HPCAR-54** | -6.903 | -82.944 | 4-C=O...Mg^2+^(2.64)  3-OH…Mg^2+^  (2.54 and 2.15)  NHC=O…Mg^2+^(2.15) | Hydrophobic: Tyr_143_,Pro_145_, Gln_146_, Gly_149_ | H-bonding: DA_17_  π-π: DC_16_ |
| **HPCAR-55** | -7.762 | -90.802 | 4-C=O...Mg^2+^(1.93)  3-OH…Mg^2+^  (2.92 and 2.26)  NHC=O…Mg^2+^(2.03) | Hydrophobic: Tyr_143_,Pro_145_, Gln_146_, Gly149, Gln_53_ | π-π: DC_16_, DA_17_ |
| **HPCAR-56** | -7.623 | -78.838 | 4-C=O...Mg^2+^(2.50)  3-OH…Mg^2+^  (2.25 and 2.18)  NHC=O…Mg^2+^(2.04) | H-bonding: Gln_146_  Hydrophobic: Tyr_143_,Pro_145_, Gln_146_ | π-π: DC_16_ |
| **HPCAR-59** | -6.680 | -68.078 | 4-C=O...Mg^2+^(2.42)  3-OH…Mg^2+^  (2.30 and 2.32)  NHC=O…Mg^2+^(2.12) | Hydrophobic: Pro_142_, Tyr_143_,Pro_145_, Gln_146_, Gly_149_ | π-π: DC_16_ |
| **HPCAR-66** | -7.623 | -78.266 | 4-C=O...Mg^2+^(2.03)  3-OH…Mg^2+^  (2.69 and 2.77)  NHC=O…Mg^2+^(2.03) | H-bonding: Gln_146_  Hydrophobic: Pro_142_, Tyr_143_,Pro_145_, Gln_146_, Gly_149_ | H-bonding: DG_4_  π-π: DC_16_ |
| **HPCAR-67** | -7.165 | -73.513 | 4-C=O...Mg^2+^(1.94)  3-OH…Mg^2+^  (2.76 and 2.28)  NHC=O…Mg^2+^(2.10) | Hydrophobic: Tyr_143_,Pro_145_, Gln_146_, Gly_149_ | π-π: DC_16_ |
| **HPCAR-69** | -7.349 | -78.275 | 4-C=O...Mg^2+^(2.25)  3-OH…Mg^2+^  (2.39 and 2.03)  NHC=O…Mg^2+^(2.03) | Hydrophobic: Tyr_143_,Pro_145_, Gln_146_, Gly_149_ | π-π: DC_16_, DA_17_ |
| **HPCAR-70** | -6.779 | -72.139 | 4-C=O...Mg^2+^(2.18)  3-OH…Mg^2+^  (2.74 and 2.38)  NHC=O…Mg^2+^(2.06) | H-bonding: Gln_146_  Hydrophobic: Gln_53_, Tyr_143_,Pro_145_, Gln_146_, Gly_149_ | H-bonding: DC_16_  π-π: DC_16_ |
| **HPCAR-75** | -6.486 | -65.578 | 4-C=O...Mg^2+^(2.11)  3-OH…Mg^2+^  (2.14 and 2.85)  NHC=O…Mg^2+^(1.92) | H-bonding: Gln_146_  Hydrophobic: Pro_142_, Tyr_143_,Pro_145_, Gln_146_, Gly_149_ | π-π: DC_16_ |
| **HPCAR-76** | -6.215 | -71.346 | 4-C=O...Mg^2+^(2.12)  3-OH…Mg^2+^  (2.87 and 2.33)  NHC=O…Mg^2+^(2.04) | Hydrophobic: Tyr_143_,Pro_145_, Gln_146_, Gly_149_ | π-π: DG_4_, DC_16_, DA_17_ |
| **HPCAR-78** | -6.708 | -65.301 | 4-C=O...Mg^2+^(2.12)  3-OH…Mg^2+^  (2.97 and 2.29)  NHC=O…Mg^2+^(2.14) | H-bonding: Glu_152_  Hydrophobic: Tyr_143_,Pro_145_, Gln_146_, Gly_149_ | π-π: DG_4_, DC_16_ |
| **HPCAR-79** | -7.681 | -72.058 | 4-C=O...Mg^2+^(2.16)  3-OH…Mg^2+^  (2.50 and 2.14)  NHC=O…Mg^2+^(2.12) | Hydrophobic: Tyr_143_,Pro_145_, Gln_146_, Gly_149_ | π-π: DC_16_ |
| **HPCAR-84** | -6.102 | -66.376 | 4-C=O...Mg^2+^(1.97)  3-OH…Mg^2+^  (2.26 and 2.20)  NHC=O…Mg^2+^(2.11) | Hydrophobic: Pro_142_, Tyr_143_,Pro_145_, Gln_146_ | H-bonding: DG_4_  π-π: DC_16_ |
| **HPCAR-86** | -6.499 | -64.845 | 4-C=O...Mg^2+^(2.61)  3-OH…Mg^2+^  (2.42 and 2.13)  NHC=O…Mg^2+^(2.05) | Hydrophobic: Tyr_143_,Pro_145_, Gln_146_ | H-bonding: DA_17_  π-π: DC_16_ |
| **HPCAR-89** | -6.648 | -75.901 | 4-C=O...Mg^2+^(2.65)  3-OH…Mg^2+^  (2.46 and 2.15)  NHC=O…Mg^2+^(2.10) | Hydrophobic: Tyr_143_,Pro_145_, Gln_146_, Gly_149_ | H-bonding: DG_4_  π-π: DC_16_ |
| **HPCAR-90** | -6.759 | -82.241 | 4-C=O...Mg^2+^(2.83)  3-OH…Mg^2+^  (2.59 and 2.25)  NHC=O…Mg^2+^(2.12) | Hydrophobic: Tyr_143_,Pro_145_, Gln_146_, Gly_149_ | H-bonding: DA_17_  π-π: DC_16_ |
| **HPCAR-91** | -6.785 | -78.789 | 4-C=O...Mg^2+^(2.12)  3-OH…Mg^2+^  (2.54 and 2.13)  NHC=O…Mg^2+^(2.05) | Hydrophobic: Tyr_143_,Pro_145_, Gln_146_, Gly_149_ | π-π: DC_16,_ DA_17_ |
| **HPCAR-92** | -6.308 | -63.519 | 4-C=O...Mg^2+^(2.12)  3-OH…Mg^2+^  (2.92 and 2.07)  NHC=O…Mg^2+^(2.01) | Hydrophobic: Tyr_143_,Pro_145_, Gln_146_, Gly_149_ | π-π: DC_16,_ |
| **HPCAR-93** | -6.586 | -67.782 | 4-C=O...Mg^2+^(2.18)  3-OH…Mg^2+^  (2.52 and 2.20)  NHC=O…Mg^2+^(2.02) | Hydrophobic: Tyr_143_,Pro_145_, Gln_146_, Gly_149_ | H-bonding: DA_17_  π-π: DC_16,_ DA_17_ |
| **HPCAR-94** | -6.151 | -62.446 | 4-C=O...Mg^2+^(1.90)  3-OH…Mg^2+^  (2.98 and 2.08)  NHC=O…Mg^2+^(2.00) | Hydrophobic: Tyr_143_,Pro_145_, Gln_146_, Gly_1496_ | π-π: DC_16_ |
| **HPCAR-95** | -6.067 | -65.127 | 4-C=O...Mg^2+^(1.90)  3-OH…Mg^2+^  (2.98 and 2.08)  NHC=O…Mg^2+^(2.00) | Hydrophobic: Tyr_143_,Pro_145_, Gln_146_, Gly_149_ | π-π: DC_16_ |
| **HPCAR-96** | -6.566 | -75.567 | 4-C=O...Mg^2+^(2.16)  3-OH…Mg^2+^  (2.51 and 2.34)  NHC=O…Mg^2+^(2.02) | Hydrophobic: Tyr_143_,Pro_145_, Gln_146_, Gly_149_ | H-bonding: DA_17_  π-π: DC_16_ |
| **HPCAR-99** | -7.655 | -67.937 | 4-C=O...Mg^2+^(1.92)  3-OH…Mg^2+^  (2.97 and 2.47)  NHC=O…Mg^2+^(1.99) | H-bonding: Gln_146_  Hydrophobic: Tyr_143_,Pro_145_, Gln_146_, Gly_149_ | H-bonding: DA_17_  π-π: DC_16_ |
| **HPCAR-100** | -6.150 | -60.280 | 4-C=O...Mg^2+^(2.19)  3-OH…Mg^2+^  (2.42 and 2.96)  NHC=O…Mg^2+^(2.06) | Hydrophobic: Tyr_143_,Pro_145_, Gln_146_, Gly_149_ | π-π: DC_16_ |
| **HPCAR-101** | -6.503 | -61.625 | 4-C=O...Mg^2+^(2.12)  3-OH…Mg^2+^  (2.92 and 2.07)  NHC=O…Mg^2+^(2.00) | Hydrophobic: Tyr_143_,Pro_145_, Gln_146_, Gly_149_ | π-π: DC_16_ |
| **HPCAR-102** | -6.306 | -69.072 | 4-C=O...Mg^2+^(2.18)  3-OH…Mg^2+^  (2.50 and 2.16)  NHC=O…Mg^2+^(2.32) | Hydrophobic: Tyr_143_,Pro_145_, Gln_146_, Gly_149_ | H-bonding: DA_17_  π-π: DC_16_ |
| **HPCAR-105** | -7.904 | -71.274 | 4-C=O...Mg^2+^(2.17)  3-OH…Mg^2+^  (2.55 and 2.14)  NHC=O…Mg^2+^(2.14) | Hydrophobic: Tyr_143_,Pro_145_, Gln_146_, Gly_149_ | π-π: DC_16_ |
| **HPCAR-108** | -6.496 | -76.504 | 4-C=O...Mg^2+^(2.11)  3-OH…Mg^2+^  (2.16 and 2.15)  NHC=O…Mg^2+^(2.73) | Hydrophobic: Tyr_143_,Pro_145_, Gln_146_, Gly_149_ | π-π: DC_16_ |
| **HPCAR-111** | -6.497 | -78.949 | 4-C=O...Mg^2+^(2.71)  3-OH…Mg^2+^  (2.15 and 2.12)  NHC=O…Mg^2+^(2.10) | H-bonding: Gln_146_  Hydrophobic: Tyr_143_,Pro_145_, Gln_146_, Gly_149_ | π-π: DC_16_, DA_17_ |
| **HPCAR-114** | -6.496 | -62.303 | 4-C=O...Mg^2+^(2.17)  3-OH…Mg^2+^  (2.25 and 2.08)  NHC=O…Mg^2+^(2.04) | H-bonding: Gln_146_  Hydrophobic: Gln_53_, Tyr_143_,Pro_145_, Gln_146_ | π-π: DC_16_ |
| **HPCAR-117** | -6.327 | -72.936 | 4-C=O...Mg^2+^(2.19)  3-OH…Mg^2+^  (2.86 and 2.00)  NHC=O…Mg^2+^(2.41) | Hydrophobic: Tyr_143_,Pro_145_, Gln_146_, Gly_149_ | H-bonding: DA_17_  π-π: DC_16_ |
| **HPCAR-118** | -6.996 | -76.313 | 4-C=O...Mg^2+^(2.16)  3-OH…Mg^2+^  (2.54 and 2.13)  NHC=O…Mg^2+^(2.13) | Hydrophobic: Tyr_143_,Pro_145_, Gln_146_, Gly_149_ | π-π: DC_16_ |
| **HPCAR-120** | -6.775 | -60.209 | 4-C=O...Mg^2+^(2.60)  3-OH…Mg^2+^  (2.50 and 2.16)  NHC=O…Mg^2+^(2.11) | H-bonding: Pro_145_  Hydrophobic: Tyr_143_,Pro_145_, Gln_146_, Gly_149_ | π-π: DG_4_, DC_16_ |
| **HPCAR-123** | -7.054 | -68.551 | 4-C=O...Mg^2+^(2.03)  3-OH…Mg^2+^  (2.71 and 2.85)  NHC=O…Mg^2+^(2.04) | H-bonding: Pro_145_  Hydrophobic: Tyr_143_,Pro_145_, Gln_146_, Gly_149_ | π-π: DC_16_ |
| **HPCAR-125** | -6.285 | -63.261 | 4-C=O...Mg^2+^(2.12)  3-OH…Mg^2+^  (2.94 and 2.14)  NHC=O…Mg^2+^(1.97) | Hydrophobic: Tyr_143_,Pro_145_, Gln_146_ | π-π: DC_16_ |
| **HPCAR-126** | -6.211 | -71.569 | 4-C=O...Mg^2+^(2.12)  3-OH…Mg^2+^  (2.82 and 2.13)  NHC=O…Mg^2+^(1.95) | Hydrophobic: Tyr_143_,Pro_145_, Gln_146_, Gly_149_ | π-π: DC_16_ |
| **HPCAR-127** | -6.693 | -63.617 | 4-C=O...Mg^2+^(1.89)  3-OH…Mg^2+^  (2.42 and 2.12)  NHC=O…Mg^2+^(2.02) | Hydrophobic: Tyr_143_,Pro_145_, Gln_146_ | π-π: DC_16_ |
| **HPCAR-128** | -6.321 | -69.135 | 4-C=O...Mg^2+^(2.17)  3-OH…Mg^2+^  (2.84 and 2.08)  NHC=O…Mg^2+^(2.67) | Hydrophobic: Tyr_143_,Pro_145_, Gln_146,_ , Gly_149_ | H-bonding: DA_17_  π-π: DC_16_ |
| **HPCAR-130** | -6.071 | -61.506 | 4-C=O...Mg^2+^(2.18)  3-OH…Mg^2+^  (2.47 and 2.29)  NHC=O…Mg^2+^(2.14) | Hydrophobic: Tyr_143_,Pro_145_, Gln_146,_ , Gly_149_ | π-π: DC_16_ |
| **HPCAR-132** | -6.192 | -62.384 | 4-C=O...Mg^2+^(1.89)  3-OH…Mg^2+^  (2.15 and 2.14)  NHC=O…Mg^2+^(2.08) | Hydrophobic: Tyr_143_,Pro_145_, Gln_146_ | π-π: DC_16_ |
| **HPCAR-134** | -7.615 | -70.750 | 4-C=O...Mg^2+^(2.17)  3-OH…Mg^2+^  (2.55 and 2.14)  NHC=O…Mg^2+^(2.14) | Hydrophobic: Tyr_143_,Pro_145_, Gln_146,_ , Gly_149_ | π-π: DC_16_ |
| **HPCAR-137** | -6.487 | -72.415 | 4-C=O...Mg^2+^(1.97)  3-OH…Mg^2+^  (2.77 and 2.25)  NHC=O…Mg^2+^(1.96) | H-bonding: Glu_152_  Hydrophobic: Tyr_143_,Pro_145_, Gln_146,_ , Gly_149_ | π-π: DG_4_, DC_16_ |
| **HPCAR-140** | -6.759 | -82.241 | 4-C=O...Mg^2+^(2.08)  3-OH…Mg^2+^  (2.87 and 2.00)  NHC=O…Mg^2+^(2.03) | Hydrophobic: Tyr_143_,Pro_145_, Gln_146,_ , Gly_149_ | H-bonding: DA_17_  π-π: DC_16_ |
| **HPCAR-142** | -6.622 | -62.706 | 4-C=O...Mg^2+^(2.18)  3-OH…Mg^2+^  (2.59 and 2.25)  NHC=O…Mg^2+^(2.12) | Hydrophobic: Tyr_143_,Pro_145_, Gln_146,_ , Gly_149_ | π-π: DC_16_ |
| **HPCAR-144** | -6.154 | -58.949 | 4-C=O...Mg^2+^(2.44)  3-OH…Mg^2+^  (2.40 and 2.11)  NHC=O…Mg^2+^(2.15) | H-bonding: Glu_152_, Gln_146_  Hydrophobic: Tyr_143_,Pro_145_, Gln_146,_ , Gly_149_ | π-π: DC_16_ |

**Table S3:** Prime/MM-GBSA output parameter values of the final selected hit compounds

| **Entry** | ΔG_bind_^a^ | ΔG_Bind Coulom_^b^ | ΔG_BindCovalet_^c^ | ΔG_Bind Hbond_^d^ | ΔG_Bind Lipo_^e^ | ΔG_BindSolvGB_^f^ | ΔG_Bind vdW_^g^ |
| --- | --- | --- | --- | --- | --- | --- | --- |
| **HPCAR-1** | -31.441 | 95.913 | 7.154 | -0.348 | -25.178 | -53.770 | -57.977 |
| **HPCAR-2** | -53.046 | -35.056 | 10.573 | -0.484 | -36.299 | 69.631 | -59.789 |
| **HPCAR-6** | -37.331 | -18.181 | 1.571 | -0.317 | -28.271 | 56.655 | -47.764 |
| **HPCAR-7** | -26.955 | -16.283 | 0.584 | -0.265 | -23.397 | 56.854 | -43.424 |
| **HPCAR-8** | -26.723 | 162.617 | 7.258 | -0.140 | -26.627 | -119.202 | -44.601 |
| **HPCAR-14** | -27.841 | 91.387 | 4.419 | -0.261 | -28.805 | -40.933 | -49.401 |
| **HPCAR-15** | -28.037 | -15.068 | 1.792 | -0.227 | -28.110 | 69.605 | -51.515 |
| **HPCAR-22** | -26.229 | -30.669 | 4.655 | -0.183 | -33.186 | 84.997 | -52.073 |
| **HPCAR-23** | -26.022 | 91.461 | 8.218 | -0.385 | -26.593 | -49.901 | -45.968 |
| **HPCAR-25** | -32.045 | -140.206 | 3.209 | -0.300 | -25.613 | 179.334 | -45.184 |
| **HPCAR-26** | -51.913 | 101.319 | 2.093 | -0.376 | -18.351 | -106.206 | -32.849 |
| **HPCAR-28** | -34.102 | 68.205 | 2.938 | -0.353 | -25.187 | -33.014 | -55.866 |
| **HPCAR-29** | -30.237 | -122.296 | 9.725 | -0.098 | -30.878 | 176.238 | -58.051 |
| **HPCAR-30** | -34.951 | -126.843 | 6.740 | -0.571 | -30.401 | 168.499 | -53.718 |
| **HPCAR-33** | -40.441 | 69.736 | 10.773 | -0.369 | -32.302 | -24.760 | -58.503 |
| **HPCAR-35** | -25.904 | 76.190 | 2.961 | -0.242 | -28.054 | -22.290 | -50.798 |
| **HPCAR-37** | -35.237 | -14.900 | 4.426 | -0.302 | -27.195 | -4.730 | -54.855 |
| **HPCAR-41** | -45.754 | -124.523 | 6.580 | -0.369 | -34.748 | 166.648 | -54.292 |
| **HPCAR-44** | -36.142 | -18.086 | 1.499 | -0.055 | -31.239 | 77.726 | -61.068 |
| **HPCAR-45** | -33.183 | 74.117 | 4.687 | -0.265 | -26.400 | -37.991 | -45.584 |
| **HPCAR-46** | -27.198 | -37.243 | 5.786 | -0.347 | -28.699 | 83.847 | -50.007 |
| **HPCAR-52** | -42.140 | 94.245 | 17.386 | -0.571 | -26.738 | -3.211 | -64.225 |
| **HPCAR-54** | -30.339 | 90.327 | 4.888 | -0.424 | -28.459 | -47.759 | -49.013 |
| **HPCAR-55** | -34.547 | 53.983 | 16.307 | -0.525 | -26.863 | -14.139 | -61.942 |
| **HPCAR-56** | -36.750 | 80.006 | 15.286 | -0.273 | -30.641 | -48.675 | -54.191 |
| **HPCAR-66** | -31.073 | 76.658 | 3.591 | -0.365 | -25.060 | -34.244 | -49.042 |
| **HPCAR-69** | -26.774 | 40.037 | 15.134 | -0.420 | -25.025 | 4.356 | -58.327 |
| **HPCAR-89** | -26.777 | 47.015 | 7.615 | -0.92 | -26.500 | 1.089 | -45.734 |
| **HPCAR-90** | -32.595 | 133.873 | 7.120 | -0.313 | -29.735 | -90.767 | -52.015 |
| **HPCAR-91** | -28.980 | 117.894 | 3.758 | -0.314 | -21.143 | -75.841 | -48.568 |
| **HPCAR-92** | -32.659 | 17.000 | 3.263 | -0.228 | -28.637 | 60.386 | -48.284 |
| **HPCAR-108** | -36.736 | 113.081 | 11.970 | -0.053 | -29.443 | -71.622 | -54.612 |
| **HPCAR-111** | -26.823 | -4.825 | 2.707 | -0.469 | -26.605 | 60.652 | -56.497 |
| **HPCAR-114** | -25.783 | -39.171 | 10.536 | -0.337 | -28.038 | 83.660 | -50.902 |
| **HPCAR-123** | -29.265 | 109.996 | 6.418 | -0.371 | -23.399 | -76.256 | -46.381 |
| **HPCAR-126** | -25.098 | 124.726 | 12.815 | -0.375 | -25.358 | -79.374 | -50.126 |
| **HPCAR-130** | -26.122 | 107.592 | 3.095 | -0.228 | -30.122 | -61.229 | -45.323 |
| **HPCAR-140** | -32.595 | 133.873 | 7.120 | -0.313 | -29.735 | -90.767 | -52.015 |
| **HPCAR-142** | -25.759 | -119.703 | 9.612 | -0.241 | -23.975 | 175.339 | -41.552 |
| **HPCAR-144** | -37.247 | -131.478 | 15.533 | -0.481 | -29.786 | 167.121 | -50.781 |

^a^ MMGBSA free energy of binding; ^b^ Contribution to the MMGBSA free energy of binding from the Coulomb energy; ^c^ Contribution to the MMGBSA free energy of binding from covalent binding; ^d^ Contribution to the MMGBSA free energy of binding from hydrogen bonding; ^e^ Contribution to the MMGBSA free energy of binding from lipophillic binding; ^f^ Contribution to the MMGBSA free energy of binding from the generalized Born electrostatic solvation energy; ^g^ Contribution to the MMGBSA free energy of binding from the van der Waals energy.


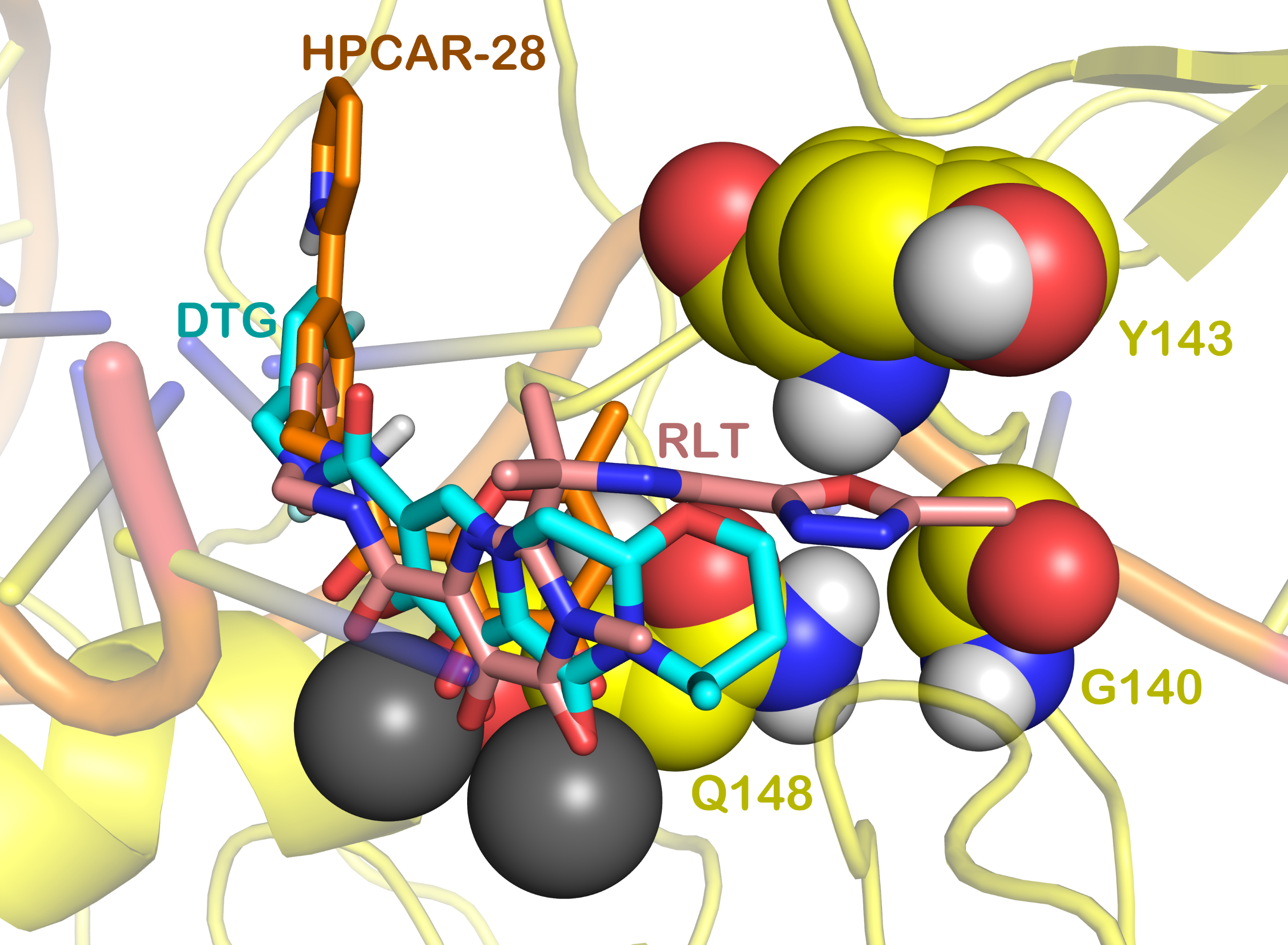


**Figure S1.** Superposition of the binding mode of **RLT**, **DTG** and **HPCAR-28** retrieved by our docking studies into the modelled HIV IN. The main residues involved in HIV-1 IN resistance and the two Mg^2+^ ions are represented as spheres. The picture was generated by PyMOL.

**^13^C-NMR**

***HPCAR-28***

**
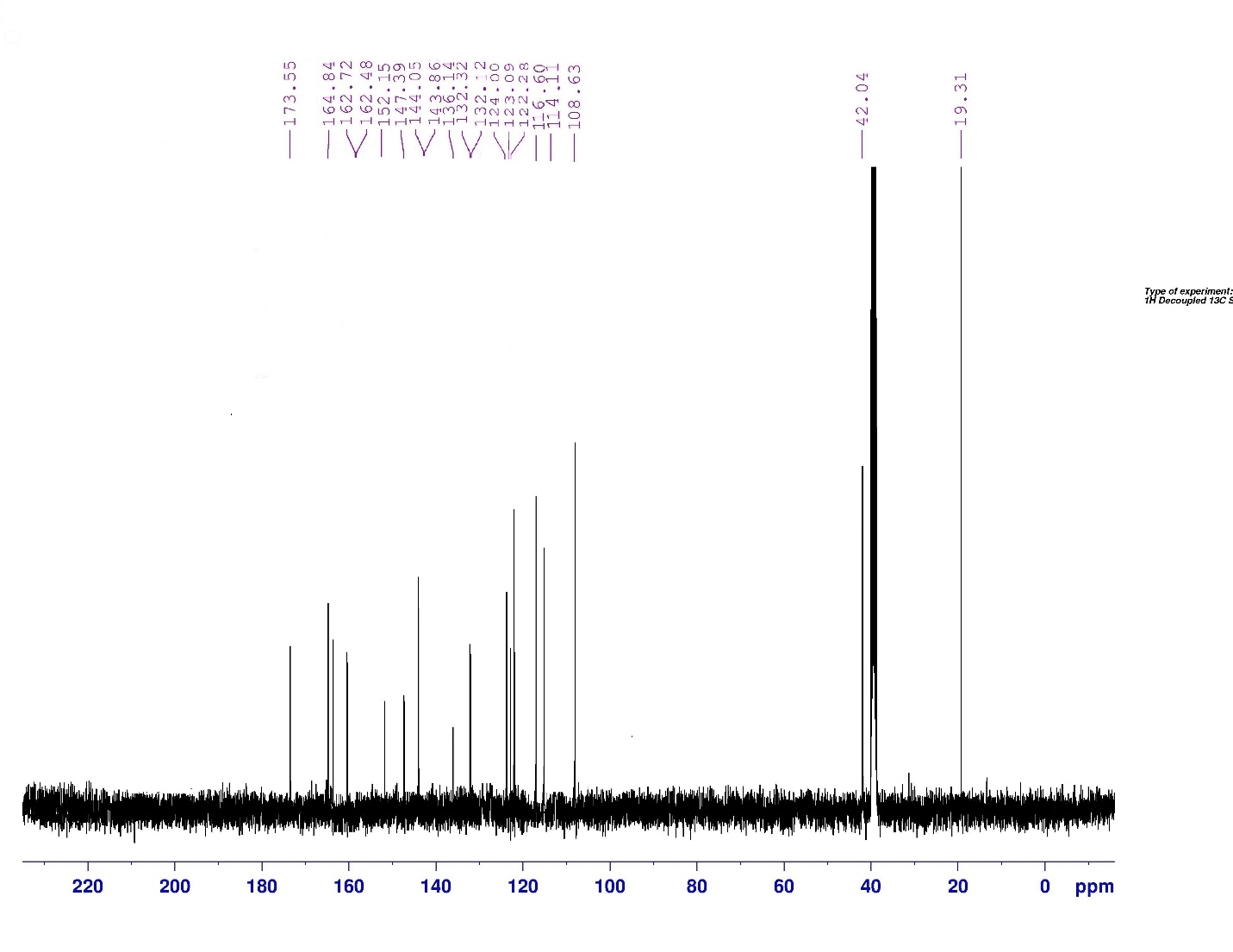
**

***HPCAR-89***

**
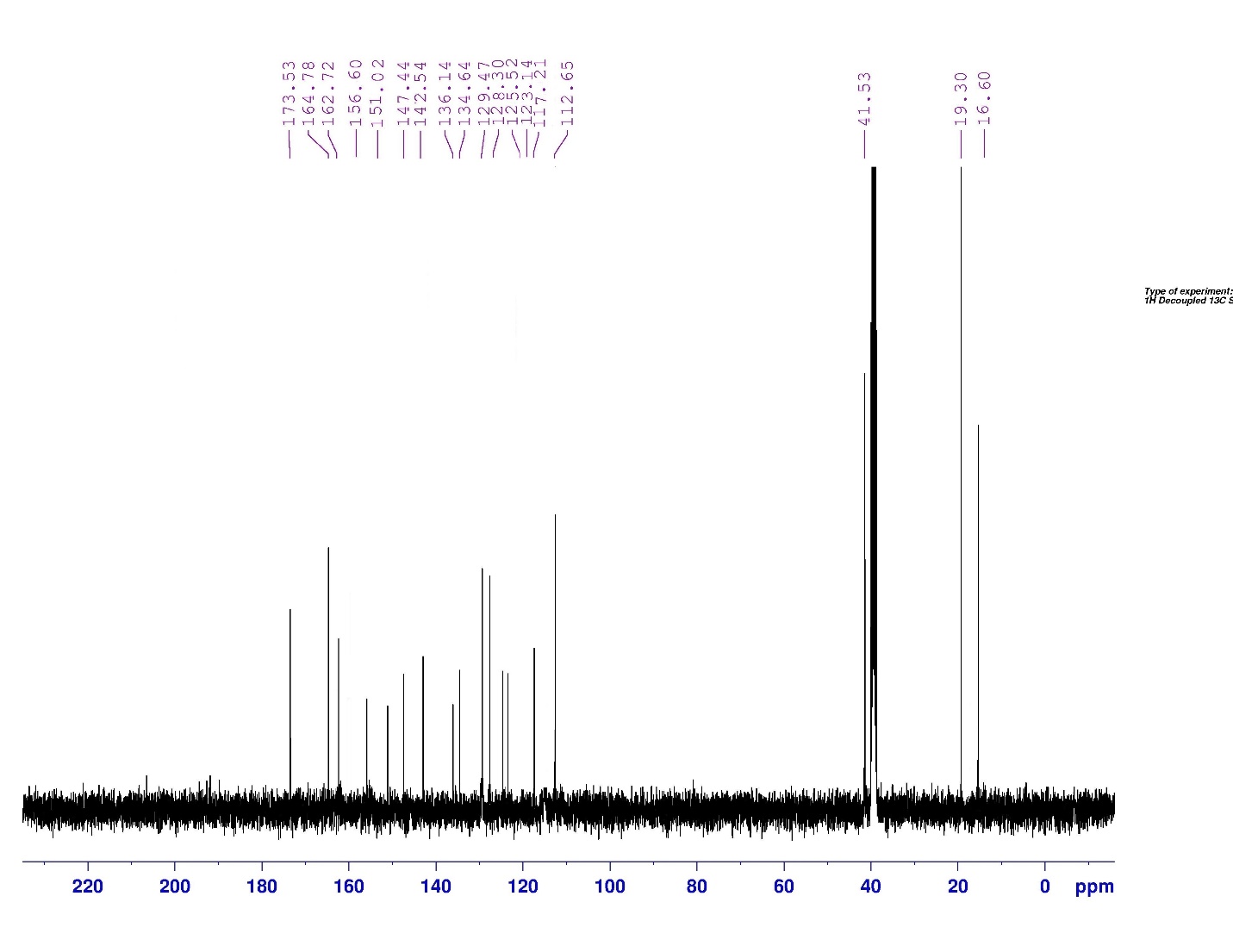
**

***HPCAR-142***

**
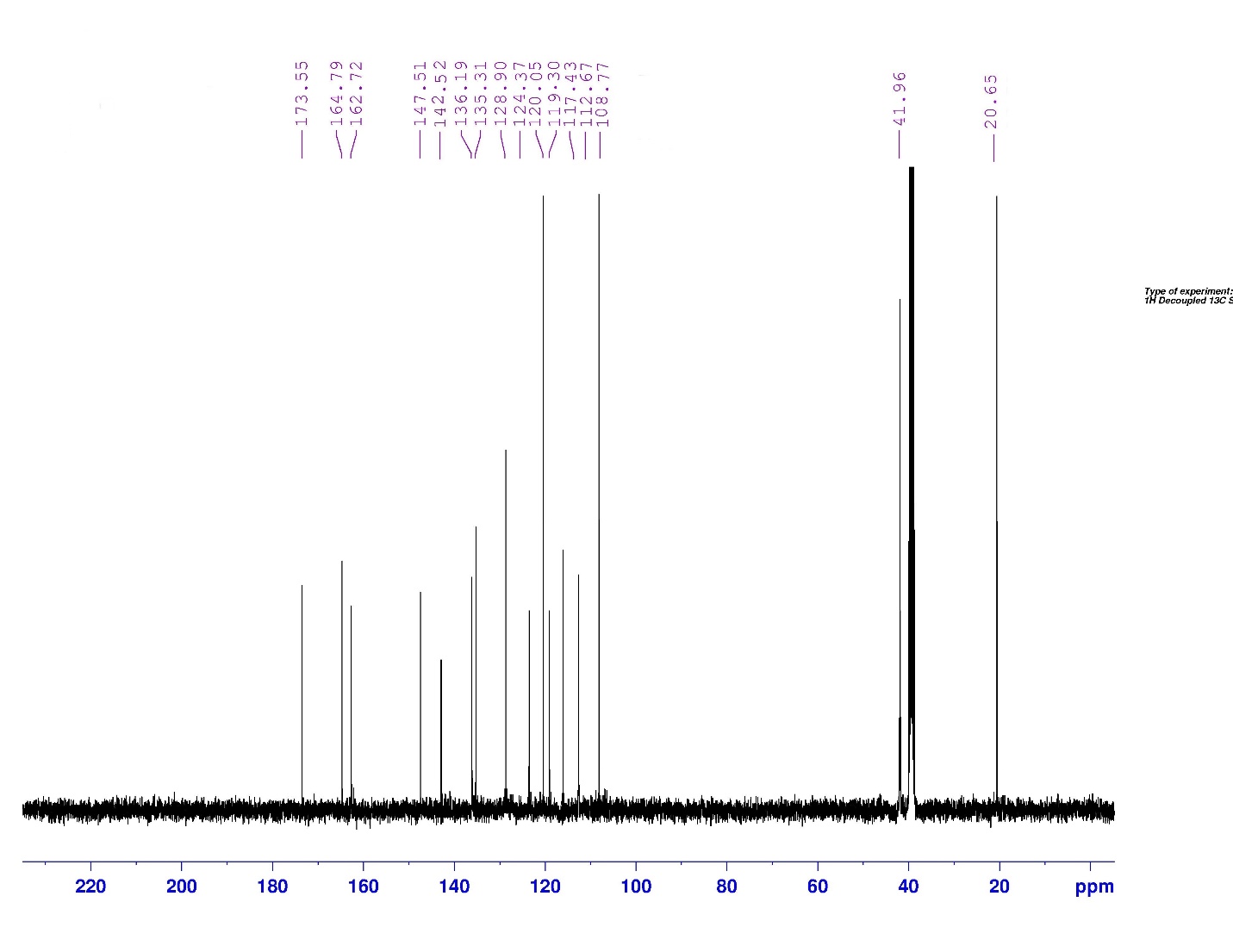
**
